# Autophagy Modulated by Inorganic Nanomaterials

**DOI:** 10.7150/thno.40414

**Published:** 2020-02-10

**Authors:** Lingling Guo, Nongyue He, Yongxiang Zhao, Tonghua Liu, Yan Deng

**Affiliations:** 1 State Key Laboratory of Bioelectronics, National Demonstration Center for Experimental Biomedical Engineering Education, School of Biological Science and Medical Engineering, Southeast University, Nanjing 210096, China; 2 Hunan Key Laboratory of Biological Nanomaterials and Devices, College of life sciences and chemistry, Hunan University of Technology, Zhuzhou 412007, Hunan, China; 3 Tibetan University of Tibetan Traditional Medicine, Lasa 850000, Tibetan, China; 4 National Center for International Bio-targeting Theranostics, Guangxi Key Laboratory of Bio-targeting Theranostics, Collaborative Innovation Center for Targeting Tumor Theranostics, Guangxi Medical University, Guangxi 530021, China

**Keywords:** inorganic nanomaterials, nanotechnology, nanotoxicity, autophagy perturbation, disease therapy

## Abstract

With the rapid development of nanotechnology, inorganic nanomaterials (NMs) have been widely applied in modern society. As human exposure to inorganic NMs is inevitable, comprehensive assessment of the safety of inorganic NMs is required. It is well known that autophagy plays dual roles in cell survival and cell death. Moreover, inorganic NMs have been proven to induce autophagy perturbation in cells. Therefore, an in-depth understanding of inorganic NMs-modulated autophagy is required for the safety assessment of inorganic NMs. This review presents an overview of a set of inorganic NMs, consisting of iron oxide NMs, silver NMs, gold NMs, carbon-based NMs, silica NMs, quantum dots, rare earth oxide NMs, zinc oxide NMs, alumina NMs, and titanium dioxide NMs, as well as how each modulates autophagy. This review emphasizes the potential mechanisms underlying NMs-induced autophagy perturbation, as well as the role of autophagy perturbation in cell fate determination. Furthermore, we also briefly review the potential roles of inorganic NMs-modulated autophagy in diagnosis and treatment of disease.

## Introduction

**Figure 1:**
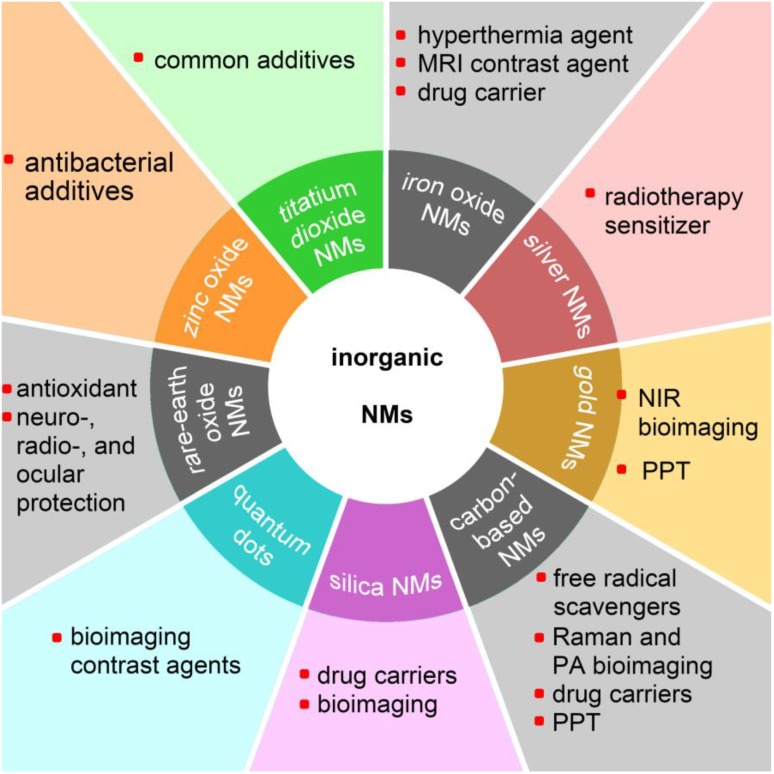
Potential applications of various inorganic nanomaterials (NMs) in the biomedical field. MRI: magnetic resonance imaging; NIR: near-infrared; PPT: photothermal therapy; PA: photoacoustic.

Nanomaterials are particulate materials with 50% or more of the constituent particles having one or more external dimensions in the size range of 1 to 100 nanometers 1. Among the engineered nanomaterials, the majority of inorganic nanomaterials (NMs) exhibit unique physicochemical and optical properties, such as that exhibited by superparamagnetic iron oxide nanoparticles (SPIONs) 2, the localized surface plasmon resonance (LSPR) effect of silver and gold nanoparticles 3, the antioxidant and free-radical scavenging capabilities of fullerenol 4, and the very high fluorescent brightness and excellent photostability of colloidal quantum dots 5. Various inorganic nanomaterials have been developed for advanced theranostics to incorporate with therapeutic and diagnostic agents in order to achieve stimuli-responsive drug release, synergetic and combinatory therapy, and multimodality therapies 6. Nanotechnology, which is generally described as the manipulation of nanoscale materials, now has a prominent role in industrial applications as well as in biomedical applications 7,8. With the rapid development of nanotechnology, NMs have been comprehensively applied in modern society. Figure 1 shows various applications of inorganic NMs in the biomedical field.

Autophagy is a natural regulated mechanism that disassembles unnecessary or dysfunctional components, thus allowing the orderly degradation and recycling of cellular components. It is well known that autophagy plays dual role in cell survival and cell death 9,10. A growing body of research has reported the ability of NMs to induce autophagy activation 11-14. It has been reported that intracellular nanoparticles are not only degraded through the endo-lysosomal pathway, but also sequestered by autophagosomes and degraded through the auto-lysosomal pathway 15,16. NMs-induced autophagy may be a cellular defensive mechanism against nanotoxicity 17, though it may also be a potential mechanism of nanotoxicity 18. Furthermore, both autophagy inhibition and activation have been reported as potent anticancer therapeutic strategies 19-27. It should be noted that in cancer therapy, autophagy has a dual-opposite role, either opposing cell transformation and progression or facilitating survival under harsh conditions and in response to chemotherapeutics.

An in-depth understanding of inorganic NMs-modulated autophagy is required for the safety assessment of inorganic NMs. This review presents an overview of a set of inorganic NMs, consisting of iron oxide NMs, silver NMs, gold NMs, carbon-based NMs, silica NMs, quantum dots, rare earth oxide NMs, zinc oxide NMs, alumina NMs, and titanium dioxide NMs, and discusses how they modulate autophagy. Special emphasis is given on the mechanism underlying the current NMs induced-autophagy perturbation and the role of autophagy perturbation in cell-fate determination. Furthermore, we also briefly review the potential roles of inorganic NMs-modulated autophagy in diagnosis and treatment of disease.

## Iron oxide nanomaterials

Iron oxide nanoparticles (IONPs) are promising materials for theranostic applications such as magnetic resonance imaging (MRI), hyperthermia, and drug delivery 28-30.

As shown in Table 1, an elevated level of autophagy is frequently observed in cells treated with IONPs. It is well known that iron ions leached from intracellular IONPs might be involved in the generation of the extremely reactive hydroxyl radical (•OH) *via* Harber-Weiss type reactions, increasing the intracellular reactive oxygen species (ROS) 31. Moreover, intracellular IONPs might also impair the function of mitochondria, enhancing the production of ROS. It has been reported that IONPs-induced increase of intracellular ROS might be a principle initiator of autophagy 32-34. Increased production of ROS can result in the damage of not only macromolecules (proteins, lipids, and nucleic acids), but also of cell organelles (e.g. mitochondria and endoplasmic reticulum) 35. One possible reason for underlying IONPs-induced autophagy is to protect cells from oxidative stress through eliminating damaged macromolecules and cell organelles caused by excessive ROS. In such a scenario, IONPs-induced autophagy can be efficiently alleviated by addition of ROS scavengers, such as N-acetyl cysteine (NAC) and natural catalase 32,34. Activation of autophagy in IONPs-treated cells might also be an attempt by cells to degrade internalized IONPs regarded as foreign materials and autophagic cargos by cells. Huang et al. 36 reported that aggregated citrate-coated IONPs induced autophagy activation in HeLa cells while no elevation of cellular ROS was observed; moreover, blocking the uptake of IONPs by dynasore, which itself does not block autophagy, led to dramatically diminished autophagic effects. Xu et al 37 reported that γ-Fe_2_O_3_ modified with polydextrose sorbitol carboxymethyl ether upregulated the expression of caveolin-1 (Cav1) in RAW264.7 cells in a time-dependent manner. Moreover, overexpression of Cav1 significantly increased LC3Ⅱ expression in macrophages and also the uptake of SPIONs by macrophages. Similarly, knockdown of Cav1 using specific siRNA markedly reduced both the uptake of SPIONs and LC3Ⅱ expression. Results demonstrated the close correlation between increased cellular uptake of IONPs and elevated autophagic activity in cells, and also indicated that enhancing degradation activity in cells in order to eliminate the internalized IONPs might be a mechanism underlying IONPs-induced autophagy activation.

Many researchers have found that the molecular mechanism underlying IONPs-induced autophagy is determined by multiple factors including cell type and physicochemical properties of IONPs. Khan et al. 32 reported that phosphorylation levels of mTOR and Akt significantly decreased while the phosphorylated AMPK significantly increased in Fe_2_O_3_-treated A549 cells, suggesting that the AMPK-mTOR-AKT signaling pathway might be involved in Fe_2_O_3_-induced autophagy. In this case, Fe_2_O_3_ NPs might affect the early phase of autophagy through initiating phagophore nucleation. However, Shi et al. 38 demonstrated that mTOR activation was not affected in OPM2 cells treated with Fe_3_O_4_ NPs, whereas expression levels of Beclin 1, Atg14, and VSP34 were increased while Bcl-2 decreased in a dose- and time-dependent manner. These results indicated that Fe_3_O_4_ NPs induced autophagy in OPM2 cells by modulating the Beclin l/Bcl-2/VPS34 complex, which plays a key role in modulating the elongation of autophagosomes. Jin et al 39 reported that two commercially available IONPs (Resovist and Feraheme, 100 μg·mL^-1^), upregulate p62 (an autophagy adapter protein that binds to ubiquitinated protein aggregates and LC3-Ⅱ) through activation of TLR4 signaling pathways, followed by phosphorylation of p38 and nuclear translocation of Nrf2. Then, p62 accumulation promotes autophagosome formation through factors necessary for aggresome-like induced structures (ALIS) formation and subsequent autophagic degradation. In this case, IONPs affected the later stage of autophagosome formation through upregulating expression of the autophagic adapter protein p62.

IONPs-modulated autophagy plays important roles in cell fate determination, as shown in Table 2. It has been reported that IONPs-modulated autophagy might play a pro-death role in cell fate 32-34. Wang et al. 34 demonstrated that carboxylate-modified α-Fe_2_O_3_ NPs (150 μg·mL^-1^) with a core size of 17 nm induced autophagic activity and cell death in PC12 cells through significantly elevating intracellular ROS in a relatively short time. However, cytotoxicity of α-Fe_2_O_3_ NPs was remarkably relieved by inhibiting autophagy at an early stage with 3-MA. A similar phenomenon was observed by Khan et al. 32, demonstrating that bare Fe_2_O_3_ NPs (100 μg·mL^-1^) with a core size of 51 nm induced autophagy and significant necrotic cell death in A549 cells through remarkable elevation of intracellular ROS. However, pre-treatment of A549 cells with 3-MA was shown to reduce the conversion of LC3-I to LC3-II and promote cellular viability. The above results imply that the pro-death role of IONPs induced autophagic activity in cell fate. However, exact mechanisms underlying IONPs-induced autophagic cell death remain unknown. While “excessive” autophagy induced by IONPs through elevating intracellular ROS over a threshold may in principle be more likely to lead to a cell death outcome, definitive experimental demonstration is lacking, and no detailed information is available on the characteristics of this so-called “excessive autophagy”. Otherwise, the disrupted autophagic process may also be an explanation of IONPs-induced pro-death autophagy, as it has been reported that Fe_3_O_4_ NPs extensively impair lysosomes, which would lead to the blockage of fusion of the autophagosome with the lysosome 40.

There are also many studies that suggest a pro-survival role of IONPs-induced autophagy in cell fate determination 37-39,41. It has been reported that polydextrose sorbitol carboxymethyl ether coated γ-Fe_2_O_3_ (200 μg·mL^-1^) with a core size of 6.5 nm induces autophagy activation in RAW264.7 cells, promoting the production of immunoregulatory cytokine IL-10 in macrophages through activation of Cav1-Notch1/HES1 signaling, leading to inhibition of inflammation in lipopolysaccharide (LPS)-induced sepsis and liver injury 37. Results indicate that the autophagic process generates pro-survival factors or activates pro-survival signaling pathways, and it is likely that IONPs induce pro-survival autophagy.

It should be noted that the effects of IONPs on autophagic activity and its role in cell fate determination should be considered together with the physicochemical properties of IONPs as well as the cell types (Table 2). It has been reported that surface modification 42, dispersity 36,43, and composition 34 of IONPs might all be important factors in IONPs-induced autophagy perturbation. In addition to physicochemical properties of IONPs, cell type is also a critical factor impacting IONPs-induced autophagy and cytotoxicity. Khan et al. 32 found that bare IONPs synthesized by themselves selectively induced autophagy in cancer cells (A549), but not in normal cells (IMR-90). Park et al. 33,44 found that γ-Fe_2_O_3_ NPs induced autophagic cell death in a murine peritoneal macrophage cell line, but not in murine alveolar macrophage cells.

IONPs-modulated autophagy exhibits a potential mechanism for anticancer therapeutics. It has been reported that IONPs exhibit anticancer effects through selectively inducing pro-death autophagy in cancer cells, but not in normal cells 32,45. It has also been reported that IONPs-induced autophagy activation exhibits a synergistic effect with chemotherapeutics to enhance cancer therapy 46.

In summary, IONPs-induced elevation of intracellular ROS may be a major initiator responsible for IONPs-induced autophagy activity. Molecular mechanisms of IONPs-modulated autophagy, as well as the role of IONPs-modulated autophagy on cell fate, should be considered together with physicochemical properties of IONPs themselves, in addition to the model cell lines. As IONPs-modulated autophagy demonstrates promise for disease treatment, comprehensive studies describing the mechanisms of IONPs-modulated autophagy are required.

## Silver nanomaterials

Sliver nanomaterials (AgNMs) not only possess broad-spectrum anti-microbial activities, but also exhibit desirable electronic, electrical, mechanical, and optical properties, and therefore have been used extensively in consumer applications 50-52. AgNMs have also been suggested as potential sensitizers for cancer radiotherapy 53,54.

AgNMs-induced autophagy perturbation has been frequently observed in a variety of cell lines, as shown in Table 1. Previous studies have shown that ROS can be generated by AgNMs owing to local surface plasmon resonance (SPR) 55. It has also been reported that AgNMs exposure caused an increase in cellular ROS, possibly due to the release of ionic silver 17. AgNMs-induced ROS increase was reported to initiate autophagy 56,57. In this case, AgNPs-induced autophagy could be efficiently inhibited by antioxidants vitamin C (Vit C) and N-acetylcysteine (NAC) 57. AgNPs might also block fusion between autophagosomes and lysosomes 58. Possible mechanisms underlying AgNPs-induced autophagic flux blockage might be AgNPs-induced lysosome dysfunction 17,58,59, disorganization of the mitochondrial network 60, and ubiquitination interference 17. Villeret et al. 60 showed that AgNPs altered mitochondrial organization and membrane potential, accompanied by increased expression of cargo-associated protein p62 and LC3-I, along with its conversion to LC3-II. Xu et al. 58 reported that AgNPs block degradation of the autophagy substrate p62 and induce autophagosome accumulation in THP-1 cells. Moreover, lysosomal impairments including alkalization and decreased membrane stability were also observed in AgNP-treated THP-1 cells. Miyayama et al. 59 reported that AgNPs induces autophagosome accumulation in A549 cells, accompanied by lysosomal pH alkalization. Moreover, p62 expression increases in a dose-dependent manner in AgNPs-treated A549 cells. The above results indicate that AgNPs treatment might result in a blockage of autophagic flux in cells; furthermore, lysosome dysfunction seems to be a primary mechanism.

Researchers have uncovered important details regarding the molecular mechanism of AgNMs-induced autophagic activity. It has been reported that levels of phosphorylated mTOR were significantly inhibited by AgNPs in Ba/F3 cells and were then restored by treatment with the antioxidants vitamin C (Vit C) and N-acetylcysteine (NAC). Results indicate that the ROS-mediated mTOR signaling pathway may be responsible for the autophagy activation induced by PVP-coated AgNPs 57. Wu et al. 61 demonstrated that specifically inhibiting ERK and JNK significantly blocks AgNPs-induced autophagy activity in U251 cells. Results indicated that PVP-coated AgNPs induced autophagy in U251 cells through modulating extracellular-signal-regulated kinase (ERK) and the c-Jun N-terminal kinase (JNK). Lin et al. 62 reported that AgNPs induced autophagy activation in Hela cells but did not alter the phosphorylation level of mTOR or its substrate, RPS6KB. Moreover, AgNPs-induced autophagy was significantly inhibited by wortmannin, an inhibitor of the PI3K pathway, suggesting that AgNPs-induced autophagy is PI3K-dependent and mTOR-independent.

AgNPs-modulated autophagy plays an important role in cell fate determination, as shown in Table 2. AgNPs-induced autophagy has been reported to be an anti-toxicity and a pro-survival process 61-64. However, mechanisms underlying the AgNPs-induced cytoprotective autophagy have rarely been studied. Lin et al. 62 reported that negatively charged, PVP-coated AgNPs (20 μg·mL^-1^) with a near spherical shape and 26 nm core size increase both the expression of LC3-I and its conversion to LC3-Ⅱ in HeLa cells through activating autophagy. Moreover, inhibition of autophagy either by chemical inhibitors or ATG5 siRNA enhances AgNPs-elicited cancer cell killing. Therefore, it was suggested that PVP-coated AgNPs induce cytoprotective autophagy in HeLa cells. Recently, it was shown that PVP-coated AgNPs activate autophagy in HeLa cells through inducing nuclear translocation of TFEB, enhancing expression of autophagy-related genes. Furthermore, the same study demonstrated that knocking down the expression of TFEB attenuates autophagy induction while enhancing cell killing in HeLa cells treated with AgNPs 64. Results indicated that TFEB was a key mediator for AgNPs-induced cytoprotective autophagy.

It has also been reported that AgNPs-induced autophagic perturbation played a pro-death role in cell fate determination 57,59,65. It has furthermore been suggested that autophagy may serve as a trigger of apoptosis 66. One possible outcome of AgNPs-induced pro-death autophagy is the activation of apoptosis. Zhu et al. 57 reported that PVP-coated AgNPs (8 μg·mL^-1^) with a near-spherical shape and core size of 11 nm induce autophagy activation in normal hematopoietic cells (Ba/F3), accompanied by DNA damage and apoptosis. Moreover, inhibiting autophagy with either a chemical inhibitor or via Atg5 silencing significantly attenuated the autophagy of AgNPs in Ba/F3 cells, as well as apoptosis and DNA damage. Results indicated that AgNPs-induced autophagy contributes to apoptosis and DNA damage, which may be the mechanism underlying AgNPs-induced pro-death autophagy. It is well known that autophagy plays a crucial role in selective removal of stress-mediated protein aggregates and injured organelles, thereby protecting cells from stress. AgNPs-induced autophagy activation may also serve as a cellular defense mechanism against nanotoxicity. However, the subsequent autophagosome-lysosome fusion defect, which leads to autophagic flux blockage, was also frequently observed in cells treated with AgNPs 58,59,65. Moreover, AgNPs-induced autophagic flux blockage was suggested as a mechanism underlying AgNPs-induced pro-death autophagy 17.

As shown in Table 2, AgNMs with different physicochemical properties can have different effects on autophagy. The documented factors that may affect AgNMs-induced autophagy include physicochemical properties of AgNMs (e.g. concentration, size, shape) and cell types. Mishra AR et al. 65 reported that PVP-coated AgNPs modulated autophagy in HepG2 cells in a concentration- and size-dependent manner. Villeret B et al. 60 reported that AgNPs-induced autophagy in BEAS-2B cells was Rab9-dependent, whereas AgNPs induced ATG-5-dependent classical autophagy in NCI-H292 cells. AgNMs-modulated autophagy also seems to be shape-dependent, as it has been reported that silver nanowires (5 *μ*g·mL^-1^) induced cytoprotective autophagy in human monocytes 67, whereas silver nanoparticles (5 *μ*g·mL^-1^) interfered with the autophagic flux in human monocytes 58.

AgNMs-modulated autophagy provides a new target for cancer therapy, as it has been observed that autophagy and apoptosis are tightly connected by common upstream signaling components 61,64. It has been reported that inhibiting AgNPs-induced autophagy leads to significantly increased cell death and effectively enhances the tumor-shrinking effect of AgNPs 62. AgNPs-induced autophagy has been reported to involve the radiosensitivity-enhancing effect of AgNPs, which may provide a useful strategy for improving the efficacy of AgNMs in cancer radiotherapy 61.

Since accumulation of AgNMs in the environment and subsequent entry into biological systems is inevitable, there are increasing bio-safety concerns related to AgNMs 68,69. Thorough investigations are still required to elucidate the mechanisms underlying AgNMs-induced autophagy perturbation and its important role in cytotoxicity.

## Gold nanomaterials

Because of their attractive physicochemical properties such as localized surface plasmon resonance, photothermal conversion, and biocompatibility 72, gold nanomaterials (AuNMs) appear to be a promising material for clinical diagnosis and treatment, including cancer cell near-infrared imaging and photothermal therapy 73, Raman signaling enhancement 74, and gene delivery 75.

AuNMs significantly increase the level of LC3-II, an autophagosome-building protein, in a variety of cell lines, as shown in Table 1. This indicates that AuNMs may induce autophagy perturbation in cells. It has been reported that AuNMs can induce autophagy, as well 76-80. Mitochondrial damage and excessive ROS generation have been suggested as possible mechanisms underlying AuNMs-induced autophagy activation. Lu et al. 78 fabricated gold nanoparticles and mesoporous silica nanoparticles into a nanohybrid (denoted GCMSNs), and they demonstrated that the presence of gold nanoparticles causes oxidative damage and mitochondrial dysfunction in A549 cells through the suppression of oxidative metabolism. Wan et al. 77 demonstrated that cetyltrimethylammonium bromide-coated gold nanorods (CTAB-GNRs) induced autophagy activation in HCT116 cells, accompanied by decreased mitochondrial membrane potential and ROS accumulation. Furthermore, CTAB-GNRs-induced autophagy activation was partially abrogated by treatment with a mitochondrial membrane potential stabilizer (cyclosporine A) or ROS scavenger (NAC). Results indicate that gold nanorods induce autophagy activation through decreasing mitochondrial membrane potential and increasing ROS generation. AuNMs can also cause impairment of autophagosome/lysosome fusion, resulting in autophagic flux blockage. Lysosome impairment caused by AuNMs treatment was reported to be a principle mechanism underlying AuNMs-induced autophagic flux blockage. Ma et al. 81 demonstrated that citrate-coated AuNPs (1 nM) were taken up into normal rat kidney cells through endocytosis, and the internalized AuNPs eventually accumulated in lysosomes and caused impairment of lysosome degradation capacity through alkalinization of lysosomal pH. Lysosome impairment made autophagosome/lysosome fusion defective, leading to autophagic flux blockage.

AuNPs-modulated autophagy can play a pro-survival role in cell fate determination. It is likely that the AuNPs-induced autophagic process generates pro-survival factors. Li et al. 82 reported that negatively charged fetal bovine serum stabilized AuNPs (1 nM) with near-spherical shape and hydrodynamic diameter of 36nm, inducing autophagosome accumulation in MRC-5 cells, accompanied by upregulation of antioxidants and stress-response proteins. Results indicate that AuNPs-induced autophagy activation might serve as a defense pathway. AuNPs-induced autophagy activation can also lead to cell death 78; however, the underlying mechanism remains unknown. AuNPs may block autophagic flux subsequently, which usually leads to cell death. It has been reported that citrate-coated AuNPs with near-spherical shape and core size of 10-50 nm cause autophagic flux blockage in normal murine kidney cells through lysosomal impairment, ultimately leading to cell death 81.

**Figure 2:**
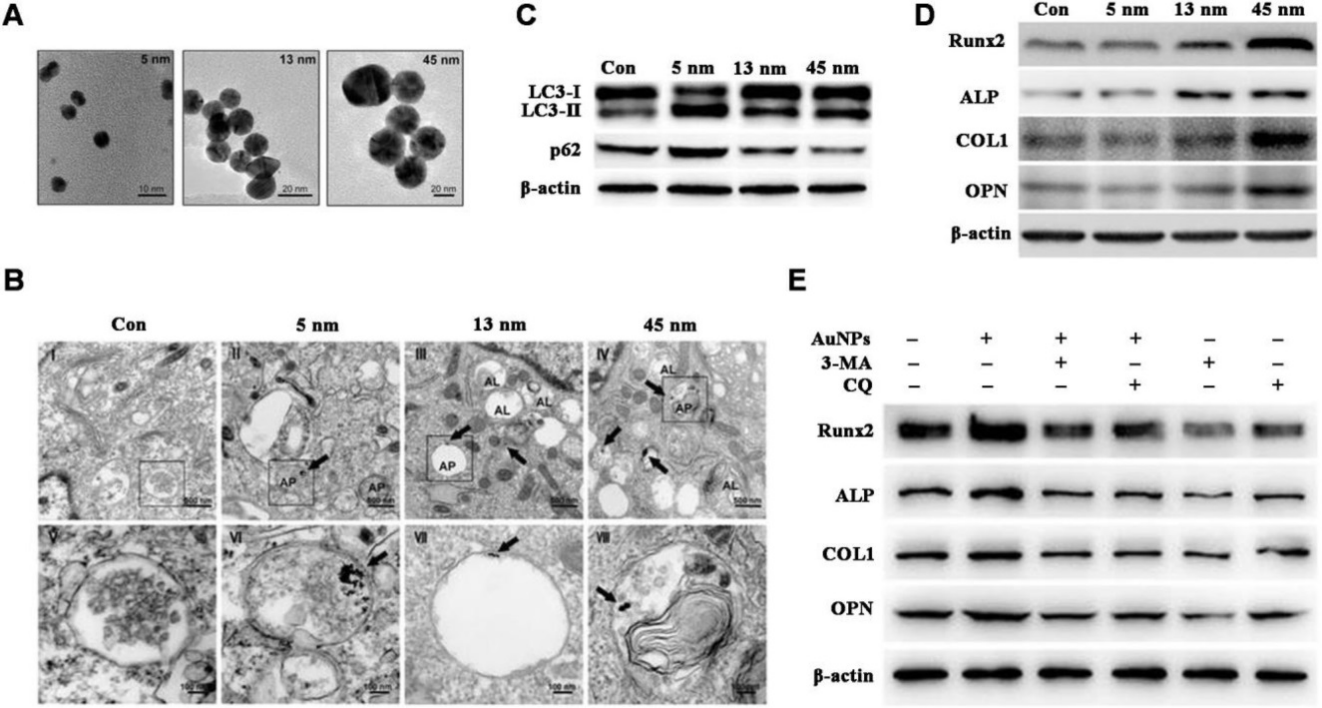
AuNPs-induced autophagy and osteogenesis is size-dependent. (A) TEM images of AuNPs of different sizes. (B-C) AuNPs-induce autophagy in PDLPs in a size-dependent manner. AP: autophagosome; AL: autolysosome. (D) AuNPs-induced osteogenesis of PDLPs in a size-dependent manner. (E) Effects of autophagy inhibitors on 45 nm AuNP-induced osteogenic differentiation. Reprinted with permission from reference 79, copyright 2017 Ivyspring International Publisher.

Documented factors impacting AuNMs-modulated autophagy include surface chemistry 76,77 and particle size 81,79. Zhang et al. 79 reported that autophagy is activated in human periodontal ligament progenitor cells (PDLPs) by 13 and 45 nm AuNPs; however, autophagy is blocked by 5 nm AuNPs, and results indicate that AuNPs-modulated autophagy is size-dependent (Figure 2A-C). Furthermore, 13 and 45 nm AuNPs not only activate autophagy in PDLPs, but also induce osteogenesis, whereas 5 nm AuNPs reduce osteogenic markers (Figure 2D). Osteogenesis induced by 45 nm AuNPs can be reversed by autophagy inhibitors (3-MA and chloroquine) (Figure 2E). Results indicate that AuNPs-modulated autophagy might be a mechanism underlying the osteogenic differentiation of PDLPs induced by AuNPs.

AuNMs-modulated autophagy appears to be a potential mechanism for cancer therapy. It has been reported that gold-silica nanohybrid-induced autophagy activation exhibits synergistic therapeutic effects with chemotherapy in A549 lung cancer xenografted nude mice 78. It has also been reported that AuNPs-modulated autophagy intensifies the TRAIL-induced apoptosis in non-small-cell lung cancer cells both *in vitro* and *in vivo*, indicating that the combination of TRAIL with AuNPs can be a potential therapeutic strategy for the treatment of non-small-cell lung cancer 80. Currently, the molecular mechanisms of AuNMs-modulated autophagy are poorly understood, and thus more investigations are required.

## Carbon-based nanomaterials

**Figure 3:**
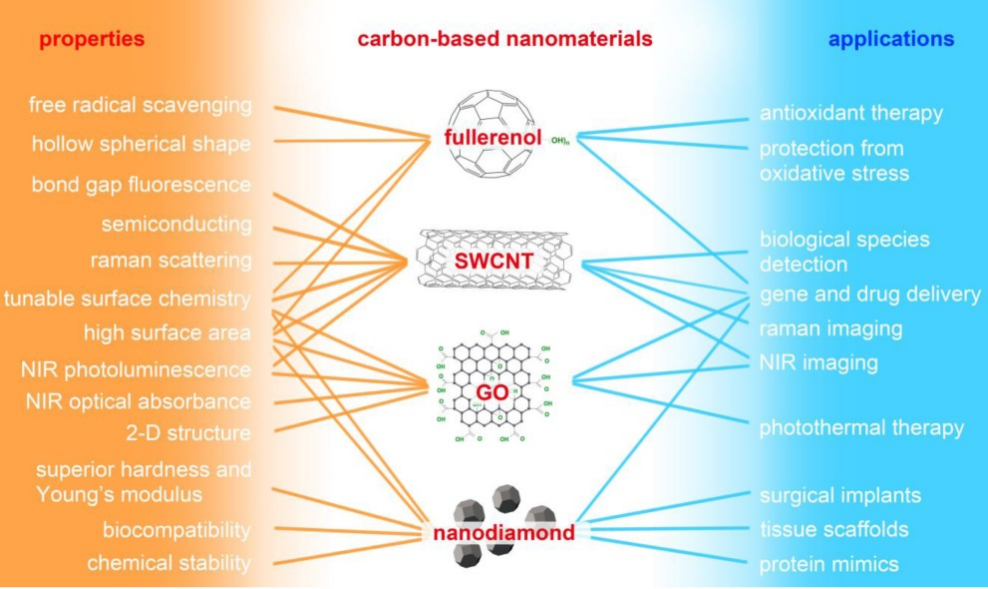
Major properties of carbon-based nanomaterials and their potential applications in biomedicine. SWCNT: single-walled carbon nanotube; GO: graphene oxide; NIR: near-infrared.

“Carbon-based nanomaterials” mainly refers to fullerene and its derivative (fullerenol), carbon nanotube (CNT), graphene oxide (GO), and nanodiamond (ND). As shown in Figure 3, carbon-based NMs possess unique physicochemical properties and have potential applications in many fields, especially biomedicine. Water-soluble fullerene derivative (fullerenol) possesses significant *in vitro* and *in vivo* antioxidant and free-radical scavenging capabilities, and it exhibits therapeutic potential against oxidative stress-associated diseases 83,84. Single-walled carbon nanotubes (SWCNT) have been widely utilized in the field of Raman and photoacoustic imaging, and drug delivery benefits from their unique structure and physicochemical properties 85,86. GO possesses unique electronic and mechanical properties as well as abundant oxygen functional groups; it demonstrates potential use in sensors, alternative energy, and biomedical applications such as bioimaging, cellular probing, drug delivery, and photothermal therapy 87-92. ND has excellent mechanical and optical properties, high surface areas, tunable surface structures, chemical stability, and biocompatibility, which make it well suited for biomedical applications such as drug delivery, tissue scaffolds, and surgical implants 93. Although carbon-based NMs appear to be promising candidates for many biomedical applications, there is a growing body of literature detailing their cytotoxic effects.

Carbon-based NMs can induce autophagy perturbation in a variety of cells, as shown in Table 1. It has been reported that carbon-based NMs can induce autophagy activation 94. Proposed mechanisms underlying carbon-based NMs-induced autophagy activation include mitochondrial dysfunction and ER stress 95, accumulation of polyubiquitinated proteins 96, and/or increased ROS generation 97. Ubiquitination of nanomaterials could also be a mechanism underlying autophagy induction by carbon-based NMs, as it has been observed that ubiquitin coats NDs involved in selective autophagy through binding to autophagy receptors 98. Carbon-based NMs can also block autophagic flux. Carbon-based NMs-induced lysosomal dysfunction and cytoskeleton disruption have been suggested as the prominent mechanism of autophagic flux blockage 85,99,100.

Exploring molecular links between carbon-based NMs and autophagy perturbation is critically important in autophagy modulation. It has been reported that the AKT-TSC2-mTOR signaling pathway is responsible for the induction of autophagy by carboxylic acid-modified CNTs in A549 cells 101. Activation of class III PI3K and MEK/ERK1/2 signaling pathways was involved in autophagy induction by GO in PC12 cells 102. Another study showed that increasing intracellular calcium ion (Ca^2+^) levels activates c-Jun N-terminal kinase (JNK), and subsequently leads to phosphorylation of Bcl-2 and dissociation of Beclin-1 from the Beclin-1-Bcl-2 complex, which was responsible for the autophagy induction by GO in HUVECs 103.

In addition to the signaling pathways mentioned above, Toll-like receptors have also been reported to play an important role in autophagy induction 104. Chen et al. 90 reported that GO treatment of RAW 264.7 cells simultaneously triggered autophagy and Toll-like receptor 4 and 9 (TLR4/TLR9)-regulated inflammatory responses, and they further demonstrated that autophagy was at least partially regulated by the TLRs pathway. Small GTPase Rab26, which regulates receptor trafficking in the cytoplasm, may be a link between TLRs and autophagy. Binotti B et al. 105 reported that Rab26 selectively localizes to presynaptic membrane vesicles and recruits both Atg16L1 and Rab33B, two components of the pre-autophagosomes. Moreover, overexpression of EGFP-tagged Rab26 induces the formation of autophagosomes in the cell bodies of hippocampal neurons. Li H et al. 106 reported that Rab26 silencing activated the TLR4 signal pathway, but that overexpression of Rab26 partially inactivated lipopolysaccharide-induced TLR4 signaling pathway. Additional research is required to clarify the role of Rab26 in TLRs-dependent autophagy.

Carbon-based NMs-modulated autophagy plays an important role in cell fate determination. It has been reported that carbon-based NMs can be a pro-survival mechanism in cells 94. A likely possibility is that carbon-based NMs-induced autophagy enhances the degradation of toxic aggregate-prone proteins (e.g. mutant huntingtin 102). However, carbon-based NMs-induced autophagy can also lead to cell death 84,95,107,96. It has been reported that PLCβ3/IP_3_/Ca^2+^/JNK signaling pathway was involved in sub-micrometer-sized GO- (SGO; 390.2 ± 51.4 nm) and nanometer-sized GO (NGO; 65.5 ± 51.4 nm)-induced autophagic cell death in endothelial cells 103. Factors affecting carbon-based NMs-modulated autophagy include surface coatings 101,108, particle size 103, and shapes 100.

Carbon-based NMs-modulated autophagy has also been exploited for disease therapy. Fullerene nanocrystals have been reported to enhance the chemotherapeutic killing of cancer cells through autophagy modulation in HeLa cells 97. Xu et al. 109 explored CaMKIIα as a regulator of fullerene C60 nanocrystals (nano-C60)-induced autophagy. They demonstrated that inhibition of CaMKIIα activity suppresses the degradation of nano-C60-induced autophagy by causing lysosomal alkalinization and enlargement, leading to enhanced cancer cell death. This investigation presented a promising strategy for improving the antitumor efficacy of nano-C60. GO effectively enhanced the clearance of mutant huntingtin (Htt), the aggregate-prone protein underlying the pathogenesis of Huntington's disease, through the activation of autophagy in GFP-Htt(Q74)/PC12 cells stably expressing green fluorescent protein-tagged Htt protein 102. Autophagic flux blockage by NDs has been reported to allosterically improve the therapeutic efficacy of arsenic trioxide (AOT)-based treatment in solid tumors 110. However, there is a lack of mechanistic data concerning the molecular links between carbon-based NMs-modulated autophagy and enhanced therapeutic effects, and thus more related studies are required.

## Silica nanomaterials

Silica nanomaterials (SiNMs) are among the most abundantly manufactured engineered nanomaterials, serving as an additive to cosmetics, drugs, printer toners, varnishes, and even food 111. Mesoporous silica nanoparticles (SiNPs) have been exploited for drug delivery, diagnosis, and bioimaging due to their high specific surface area and pore volume, tunable pore structures, and excellent physicochemical stability 112-116. With the growing applications of SiNMs, there are growing concerns about their potential hazards to human health. It has been reported that autophagy induction may attenuate cytotoxicity caused by SiNPs, as it has been reported that dioscin promoting autophagy in alveolar macrophages relieved crystalline-silica-stimulated ROS stress and facilitated cell survival 117.

As shown in Table 1, SiNPs induces autophagy perturbation in a variety of cell lines. SiNPs can also induce autophagy activation. Mechanisms underlying SiNPs-induced autophagy activation include cytoskeleton disruption 118, oxidative stress 119, ER stress 120, and mitochondrial damage 121. It has also been reported that SiNPs can block autophagic flux through lysosome impairment 122.

The PI3K/AKT/mTOR pathway was reported to be involved in surface negatively charged silica NPs-induced autophagy activation in HUVECs 123. It has also been reported that activation of the EIF2AK3 and ATF6 UPR pathways is responsible for autophagosome accumulation by silica NPs in L-02 cells 120. In another case, it was reported that autophagy induction by PEGylated silica-based NPs in MC3T3-E1 cells was dependent on the mitogen activated protein kinase ERK1/2 124.

SiNMs-modulated autophagy plays dual roles in cell survival and cell death. One study showed that autophagy induction by bioactive SiNPs promoted *in vitro* differentiation and mineralization of murine pre-osteoblasts (MC3T3-E1) 124. It was reported that SiNPs enhanced autophagic activity in HUVECs, accompanied by cellular homeostasis disruption and angiogenesis impairment 118. It has also been reported that SiNPs can block autophagic flux, which usually leads to cell death. Wang et al. 122 reported that SiNPs induce increased LC3B-Ⅱ expression in hepatocytes in a dose- and time-dependent manner, in accordance with SiNPs-induced cytotoxicity in hepatocytes. However, p62 degradation was not observed in hepatocytes at any dose of SiNPs at any time. After treating with bafilomycin A1 (BafA1), which suppresses fusion between autophagosomes and lysosomes, LC3B-Ⅱ expression increases in hepatocytes treated with lower doses of SiNPs, whereas p62 expression increases only in cells exposed to lower doses of SiNPs. Furthermore, higher-dose SiNPs treatment caused lysosomal destruction, lysosomal cathepsin expression downregulation, and increased lysosomal membrane permeability. Results indicate that high-dose SiNPs inhibits autophagosome degradation via lysosomal impairment in hepatocytes, resulting in autophagy dysfunction.

Mesoporous silica NPs significantly sensitize doxorubicin for killing cancer cells by increasing ROS generation and triggering the mitochondria-related autophagic lysosome pathway 125. Results indicate that silica NMs-modulated autophagy may also be exploited for cancer therapy.

## Quantum dots

Quantum dots (QDs) are nanoscale (2-10 nm) fluorescent colloids composed of semiconductor materials, commonly used as fluorescent probes for bioimaging fixed cells and tissues 5. It has also been reported that QDs have the potential to be used as multimodal contrast agents during drug delivery 126 and in bioimaging 127. However, precautions should be taken when QDs are used *in vivo*, as leaking of toxic core metals from QDs is able to generate ROS, which damage cellular membrane integrity, and inflict oxidative damage on intracellular DNA, proteins, and lipids 128,129.

QDs can induce autophagy perturbation in cells, as shown in Table 1. QDs-caused oxidative stress has been reported to be responsible for QDs-induced autophagy 121,130. QDs-modulated autophagy plays important roles in cell fate determination. It was reported that QDs-induced autophagy activation in a murine renal adenocarcinoma cell line is a defensive/survival mechanism against nanotoxicity 130. QDs-induced autophagy can also play a pro-death role in cell fate determination 5,121. It has been reported that elevated autophagy is at least partially responsible for the *in vivo* synaptic dysfunction induced by CdSe/ZnS QDs 131.

## Rare earth oxide nanomaterials

Rare earth elements are a category of materials including 17 different members with similar chemical properties. Cerium is one of the rare earth elements that belongs to the lanthanide series. Cerium oxide (CeO_2_) is routinely used in polishing glass and jewelry, and it is also used in catalytic converters for automobile exhaust systems and other commercial applications 132. Cerium oxide nanoparticles (NPs) are promising for therapeutic applications including antioxidant therapy, neuroprotection, radioprotection, and ocular protection 132-135. Because of their clinical application prospects, the biosafety of rare earth oxide nanomaterials (REO NMs) is drawing increased attention. Cerium oxide NPs at relatively low doses have been reported to cause mitochondrial damage, overexpression of apoptosis-inducing factor, and autophagy induction in human peripheral blood monocytes 136. REO NMs can induce autophagy perturbation in a variety of cell lines, as shown in Table 1.

Neodymium is one of the rare earth elements that belong to the lanthanide series, as well. Autophagy induction by neodymium oxide NPs is accompanied by cell cycle arrest in S-phase, mild disruption of mitochondrial membrane potential, and inhibition of proteasome activity, as observed in non-small cell lung cancer cells (NCI-H460) 137. Another study reported that autophagy, induced by cerium oxide NPs through promoting activation of the transcription factor EB, promotes clearance of proteolipid aggregates in fibroblasts derived from a patient with late infantile neuronal ceroid lipofuscinosis 138. Zhang et al. 139 reported that lanthanide-based upconversion nanoparticles (UCNs) are able to induce obvious autophagy and hepatotoxicity in mouse liver; furthermore, they demonstrated that coating with specific peptide RE-1 reduced autophagy and hepatotoxicity of UCNs. Zhu et al. 140 demonstrated that UCNs induced pro-death autophagy in Kupffer cells and liver injury. Furthermore, inhibition of autophagy enhances Kupffer survival and further abrogates UCN-induced liver toxicity. Recently, Zhang et al. 141 revealed the detailed mechanisms of UCNs-induced liver damage: insufficient PIP5K1B on the autolysosome membrane after treatment with UCNs causes disrupted phospholipid transition from PI(4)P to PI(4,5)P2 on the enlarged autolysosome membrane. This subsequently leads to clathrin recruitment failure and causes persistent, large autolysosomes in hepatocytes, which finally lead to hepatotoxicity.

Autophagic flux defect is caused by a series of rare-earth oxide NPs including La_2_O_3_, Gd_2_O_3_, Sm_2_O_3_, and Yb_2_O_3_ through lysosomal dysfunction, which disrupts homeostatic regulation of activated NLRP3 complexes, as has been observed in a myeloid cell line (THP-1) 142. Wei et al. 143 demonstrated that europium hydroxide nanorods (EHNs)-induced autophagy enhances the degradation of mutant huntingtin protein aggregation in Neuro2a cells. Afterwards, they revealed that EHNs-induced autophagy does not follow the classical AKT-mTOR and AMPK signaling pathways, but instead the MEK/ERK1/2 signaling pathway. Furthermore, they demonstrated that the combined treatment of EHNs and the autophagy inducer trehalose led to more degradation of mutant huntingtin protein aggregation, suggesting that enhanced clearance of intracellular protein aggregates may be achieved through combined treatment with two or more autophagy inducers. This information is vital for the treatment of diverse neurogenerative diseases 144.

## Zinc oxide nanomaterials

Zinc oxide nanomaterials (ZnO-NMs) have been extensively used in many dental materials, cosmetic products, and textiles because of their antibacterial performance and ultraviolet light-absorbing properties. Zinc oxide nanoparticles (ZnO-NPs) are also versatile platforms for biomedical applications and therapeutic intervention 145,146. However, biosafety concerns have been raised over the wide applications of ZnO-NPs. Cellular zinc homeostasis disruption, ROS generation, mitochondrial damage, and autophagy induction have been reported to be caused by zinc oxide nanoparticles 147-149.

Recently, Hu et al. 150 investigated the subcellular mechanism of pro-death autophagy elicited by ZnO-NPs. The group demonstrated that the acceleration of zinc ion release by autophagy and the sequentially increasing intracellular ROS generation in cancer cells contributed to cell death. Furthermore, they demonstrated that combinatory use of ZnO-NPs and doxorubicin results in sensitizing the chemotherapeutic killing of both normal cancer cells and drug-resistant cells through autophagy-mediated intracellular dissolution of ZnO-NPs. These results indicate that the modulation of autophagy holds great promise for improving the efficacy of tumor chemotherapy.

ZnO-NMs-modulated autophagy is closely correlated with cytotoxicity. It has been reported that autophagy induction by ZnO-NPs ultimately leads to autophagic flux blockage in A549 cells through lysosomal impairment, which is caused by the enhanced dissolution of zinc oxide NPs and release of zinc ions, decreasing cell viability and causing cell death 151. Autophagy modulated by ZnO-NPs may be dependent on particle size, as it has been reported that 50 nm ZnO-NPs interfered with the autophagic flux in A549 cells and led to cell death, whereas 200 nm ZnO-NPs failed to induce autophagy-mediated toxicity 152.

## Alumina and titanium dioxide nanomaterials

Nano-sized alumina (Al_2_O_3_) and titanium dioxide (TiO_2_) are among the most abundantly manufactured engineered nanomaterials. Titanium dioxide is a common additive in food, personal care items, and other consumer products 153. Therefore, one can predict that many workers around the world will encounter Al_2_O_3_ and TiO_2_ NMs, and thus occupational exposures can be anticipated. Cellular exposure to TiO_2_ NPs resulted in ROS production, DNA damage, and autophagy induction, as has been observed in human cerebral endothelial cells (HCECs) 47. Prolonged exposure (72 h) to TiO_2_ NPs was found to cause autophagic flux blockage in H4/a-syn-GFP cells, whereas short exposure (24 h) to TiO_2_ NPs promoted autophagic flux 154. Autophagy induction seems to be an important mechanism involved in Al_2_O_3_ NMs-induced toxicity, as has been observed in human cerebral microvascular endothelial cells (HCMECs) 155 and RAW 264.7 cells 156.

Besides its adverse effects, autophagy induction by nanosized Al_2_O_3_ also exhibits potential applications in the biomedical field. Autophagy induction by Al_2_O_3_ NPs inhibits the activation of osteoclasts and thus reduces osteolysis and aseptic loosening by decreasing the expression of RANKL 157. α-Al_2_O_3_ NPs-modulated autophagy efficiently enhances antigen cross-presentation, a key step for the successful development of therapeutic cancer vaccines, through delivering significant amounts of antigens into autophagosomes in dendritic cells, which then present the antigens to T cells through autophagy 158.

## Conclusion and perspective

**Figure 4:**
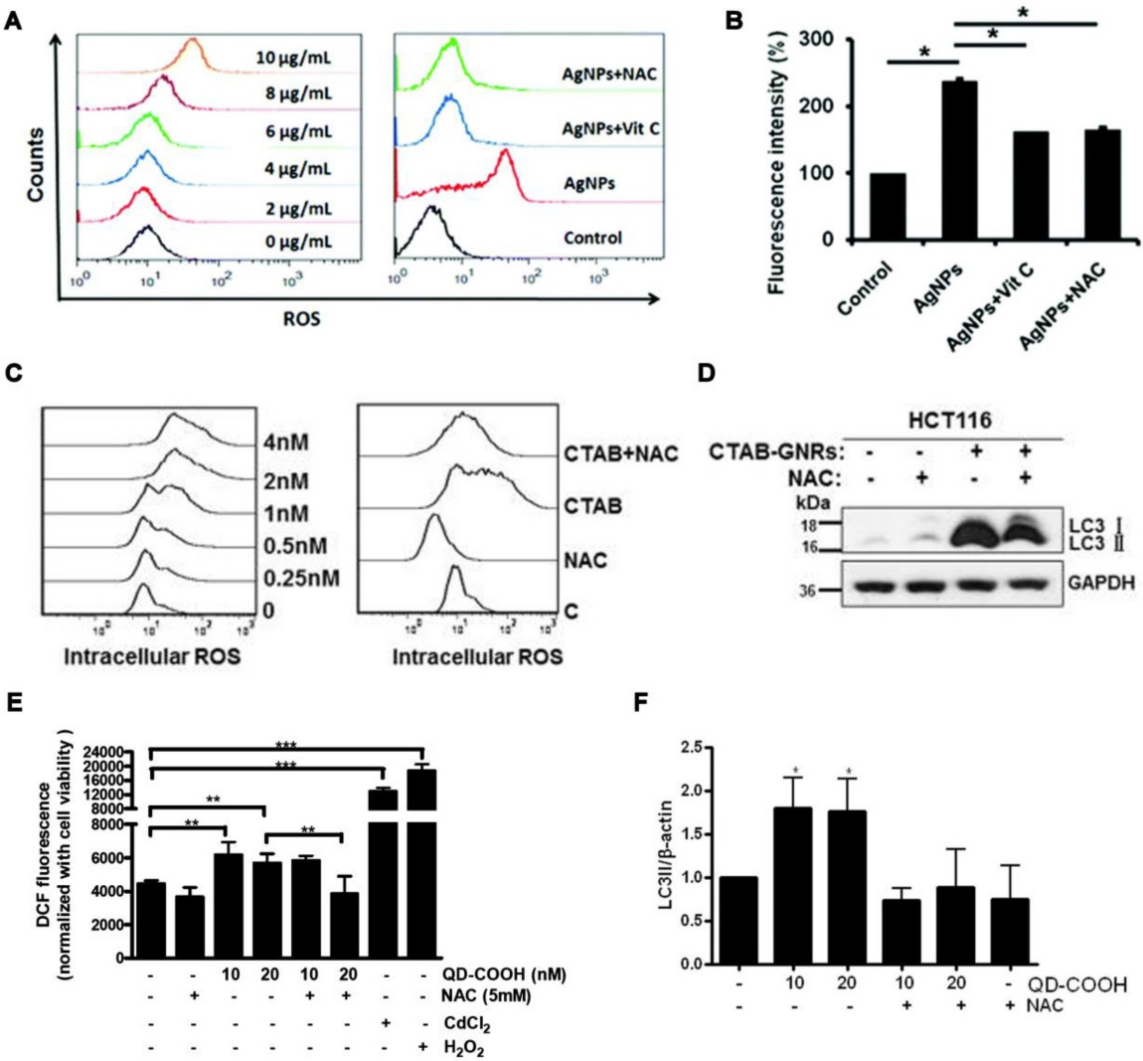
Increased intracellular ROS generation and its role in inorganic nanomaterials-modulated autophagy. (A) Effect of AgNPs on the production of ROS, and (B) effect of the ROS scavengers Vit C and NAC on reduction in cell autophagy induced by AgNPs detected by LysoTracker Red assay. Reprinted with permission from reference 57, copyright 2017 Royal Society of Chemistry. (C) Effect of gold nanorods (CTAB) on the production of ROS, and (D) effect of NAC on the reduction in cell autophagy induced by gold nanorods (CTAB-GNRs) detected by western blot assay. Reprinted with permission from reference 77, copyright 2015 Springer Nature. (E) Effect of quantum dots (QD-COOH) on intracellular ROS determined using 2,7-dichlorofluorescein diacetate, and (F) effect of NAC on the reduction of cell autophagy induced by QD-COOH, detected by western blot. Reprinted with permission from reference 130, copyright 2013 American Chemical Society.

**Figure 5:**
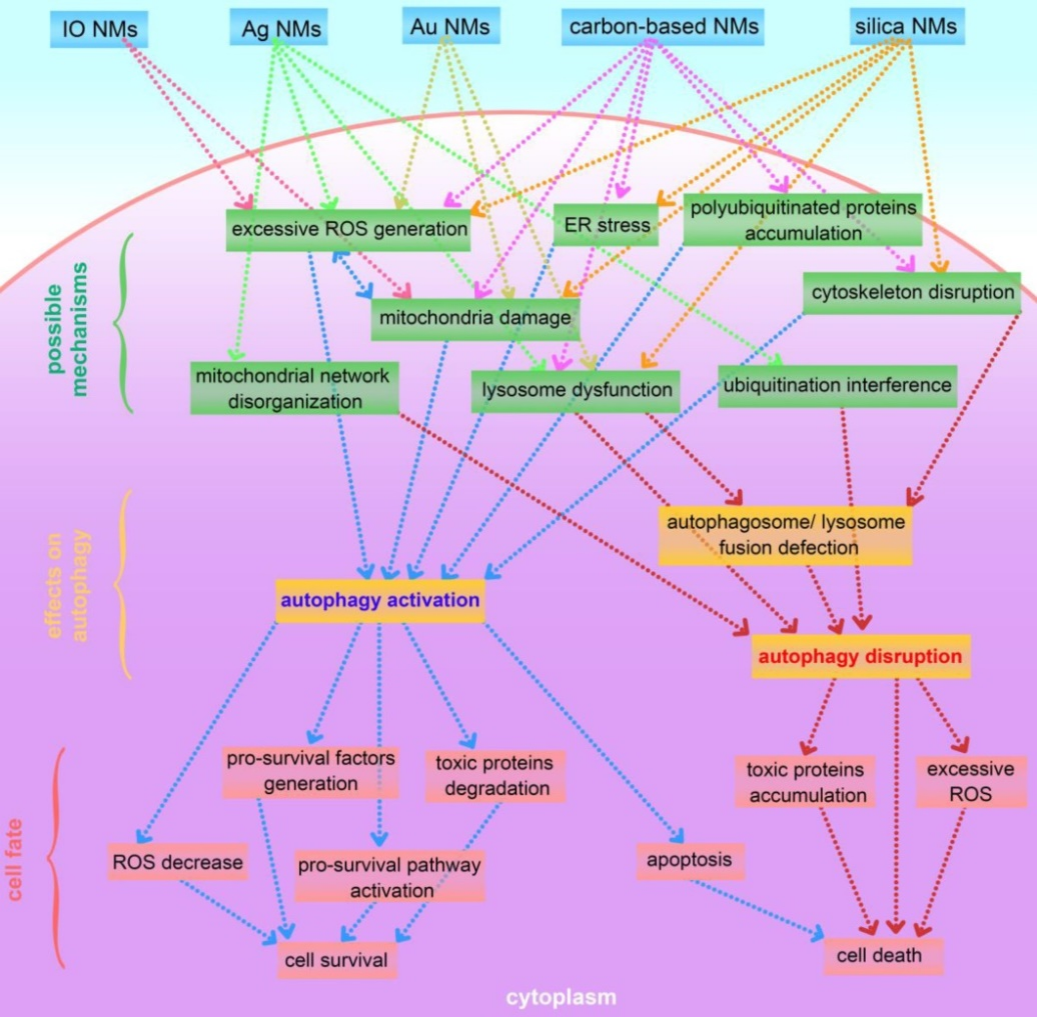
A summary of possible mechanisms underlying inorganic nanomaterials-modulated autophagy, and important roles of autophagy in cytotoxicity. IO NMs: iron oxide nanomaterials; Ag NMs: silver nanomaterials; Au NMs: gold nanomaterials.

This review presents an overview of a set of inorganic nanomaterials (NMs) including iron oxide nanomaterials, silver NMs, gold NMs, carbon-based NMs, silica NMs, quantum dots, rare earth oxide NMs, zinc oxide NMs, alumina NMs, and titanium dioxide NMs, and discusses how each modulates autophagy and of their role in cell fate determination. As shown in Figure 4, inorganic nanomaterials including AgNMs, AuNMs, and quantum dots, are frequently observed to elevate intracellular ROS generation, accompanied by autophagy activation. Furthermore, ROS scavengers (e.g. NAC) can efficiently suppress inorganic nanomaterials-induced autophagy. Therefore, inorganic NMs-induced increased ROS generation may be a prominent mechanism underlying IONPs-induced autophagy activation. In addition to excessive ROS generation, there are several other mechanisms responsible for inorganic NMs-modulated autophagy, including mitochondria damage, endoplasmic reticulum (ER) stress, polyubiquitinated protein accumulation, cytoskeleton disruption, mitochondrial network disorganization, lysosome dysfunction, and ubiquitination interference. Several possible mechanisms underlying inorganic nanomaterials-modulated autophagy are summarized in Figure 5. As shown in Figure 5, inorganic NMs causing excessive ROS generation, mitochondrial damage, ER stress, and polyubiquitinated protein accumulation are more likely to induce autophagy activation, while mitochondrial network disorganization, lysosome dysfunction, cytoskeleton disruption, and ubiquitination interference tend to block autophagic flux, resulting in autophagy disruption. Furthermore, Figure 5 summarizes the possible roles of inorganic NMs-modulated autophagy in cell fate determination. Inorganic NMs-induced autophagy activation may promote cell survival by decreasing intracellular ROS, generating pro-survival factors, degrading toxic proteins, or activating pro-survival pathways. Inorganic NMs-induced autophagy activation may also lead to cell death through promoting apoptosis. However, inorganic NMs-induced autophagy disruption usually results in cell death through toxic protein accumulation and excessive ROS. It should be noted that the effects of IONPs on autophagic activity and their role in cell fate determination should be considered together with the physicochemical properties of IONPs, as well as the cell types.

Inorganic NMs-modulated autophagy provides a new target for therapy. Inorganic NMs-modulated autophagy has been reported to play an important role in radiotherapy and chemotherapy sensitization, and in promoting the clearance of huntingtin protein aggregation in neurons, indicating that it can be a potential tool for therapy. However, as the research on autophagy modulation by nanomaterial is still at a rudimentary stage, many scientific questions remain largely unanswered. Molecular links between inorganic NMs-modulated autophagy and enhanced therapeutic effects remain murky. Therefore, comprehensive investigations are still required to fully explore the values of inorganic NMs-modulated autophagy for theranostic application.

## Figures and Tables

**Table 1:** Inorganic NMs-modulated autophagy was frequently observed in a variety of cell lines.

NMs	Cell line
IO NMs	A549 32, RAW264.7 33,37,39, PC12 34, HeLa 36, OPM2 38, MCF-7 40, human monocytes 41, SKOV-3 42, OECM1 45, HepG2 46, Human cerebral endothelial cells 47, U2OS 48, Mouse dendritic cells 49
Ag NMs	NIH 3T3 70, U251 56, 61, T24 71, NCI-H292 60, THP-1 monocyte 58,67, HepG2 65, A549 59, HeLa 62, 64, Ba/F3 57
Au NMs	HUVECs 76, HEK293T 77, L02 77, HFF 77, HCT116 77, BEL7402 77, PC3 77, A549 78, NRK 81, MRC-5 82, human periodontal ligament progenitor cells 79, Calu-1 80
Carbon-based NMs	LLC-PK1 84, HUVECs 85,96,103, RAW264.7 90,100,95, A549 99,101, BEAS-2B 100, SK-N-SH 94, HeLa 97, PC12 102, HEK 293 108, U87 108, 143B 109, MG63 109
Silica NMs	HUVECs 118,123,119, L-02 122,120, HepG2 122
Quantum dots	LLC-PK1 5, murine embryonic fibroblast 121, RAG 130, hippocampal neurons 131, HeLa 131
Rare earth oxide NMs	NCI-H460 137, late infinite neuronal ceroid lipofuscinosis fibroblasts 138, HeLa 139, Kupffer 140, primary hepatocytes 141, THP-1 142, Neuro 2a 143
Zinc oxide NMs	HeLa 150, A549 151,152
Alumina NMs	human cerebral microvascular endothelial cells (HCMECs) 155, RAW264.7 156, T cells 158
Titanium dioxide NMs	human cerebral endothelial cells (HCECs) 47, H4/a-syn-GFP 154

**Table 2:** Inorganic nanomaterials-modulated autophagy and its effects on cell fate.

NMs	Size (characterization method); Zeta Pot.; shape or dispersity	Coating	Concentration	Exposure period	Model cells	Mechanism	Cell fate	Ref.
IONPs	51 nm (TEM); -39 mV; aggregates	Bare	100 μg·mL^-1^	48 h	A549 cells	ROS upregulation and p-mTOR expression inhibition	Cell death	32
Fe_3_O_4_	41 nm (DLS); -51 mV; near spherical	Phospholipid	50 μg·mL^-1^	24 h	RAW264.7 cells	--	Apoptotic cell death	33
α-Fe_2_O_3_ NPs	17 nm (TEM); near spherical	Caboxylate	150 μg·mL^-1^	24 h	PC12 cells	ROS upregulation	Cell death and growth arrest	34
γ-Fe_2_O_3_	6.5 nm (TEM); --; nano-aggregates	polydextrose sorbitol carboxymethyl ether	200 μg·mL^-1^	24 h	RAW 264.7	Activation Cav1-Notch1/HES1 pathway	Cell survival	37
Fe_3_O_4_ NPs	>10 nm (TEM); 22 mV, -29 mV, or 5 mV; near spherical	Bare, DA, DMSA, or DA-PAA-PEG	100 μg·mL^-1^	9 h	OPM2 cells	upregulation of Beclin l/Bcl-2/VPS34 complex	Cell survival	38
Resovist and Feraheme	62 nm (DLS), 30 nm (DLS); --; --	Carboxydextran, polyglucose sorbitol carboxymethyl ether	100 μg·mL^-1^	24 h	RAW 264.7	Activation TLR4-p38-Nrf2-p62 pathway	Cell survival	39
IO-NPs	60 nm (DLS); -11 mV; nano-aggregates	Dextran	100 μg·mL^-1^	24 h, 48 h	Human monocytes	--	Cell survival	41
AgNPs	11 nm (TEM); near spherical	PVP	8 μg·mL^-1^	24 h	Ba/F3 cells	ROS activation and p-mTOR inhibition	Apoptosis	57
AgNPs	>30 nm (TEM); -4.3 mV; near spherical shape	--	5 and 10 μg·mL^-1^	48 h	THP-1 cells	Lysosome dysfunction	Imedence of PMA-induced monocyte differentiation	58
AgNPs	70 nm (DLS); -31 mV in culture medium; near spherical	Citrate	50, 100, and 200 μg·mL^-1^	24 h	A549 cells	Lysosome dysfunction	Cell death	59
AgNPs	27 nm (TEM); -13 mV; near spherical	PVP	20 μg·mL^-1^	24 h	Hela cells	--	Promoted cell survival	62
AgNPs	27 nm (TEM); --; near shperical	PVP	10 μg·mL^-1^	8 h	HeLa cells	nucleus translocation of TFEB	Cell survival	64
AgNPs	14 nm, 52 nm, and 102nm (TEM); spherical	PVP	10 μg·mL^-1^	12 h, 24 h	HepG2 cells	--	Apoptosis	65
Au naorods	100 nm length and 4 aspect ratio (TEM); 38 mV; nanorod	CTAB	2 nM	24 h	HCT116 cells	ROS upregulation	Apoptosis	77
AuNPs	18 nm, 55 nm, and 84 nm (DLS); negative; near spherical	--	50 μg·mL^-1^	24 h	Calu-1 cells	Mitochondrial dysfunction	Cell death	80
AuNPs	10 nm, 25 nm, and 50 nm (TEM); negative; near spherical	Citrate	1 nM	24 h	NRK cells	--	--	81
AuNPs	36 nm (DLS); -11 mV; near spherical	Fetal bovine serum	1 nM	72 h	MRC-5 cells	Oxidative stress	Cell survival	82
C_60_(OH)_x_	15.7 nm (DLS); -49 mV; nano-aggregates	--	6 mM	6 h, 24 h	LLC-PK1 cells	--	Cell death	84
MWCNT	60 nm diameter; -42 mV; nanotube	Carboxylated	100 μg·mL^-1^	24 h	HUVECs	--	Apoptosis	85
GO	350 nm diameter, 1.0- 1.2 nm thickness (AFM); nanosheets	--	100 μg·mL^-1^	24 h	RAW 264.7 cells	Activation TLR signaling cascades	Cell death	90
GO	100 nm-2 μm diameter, 1 nm thickness (SEM); negative; nanosheet	--	8 mg·mL^-1^	12 h	SK-N-SH cells	--	Promoted neuro cell survival	94
Graphite carbon nanofibers	79 nm outer and 7 nm inner diameter (TEM); -30 mV; nanofiber	--	25 μg·mL^-1^	24 h	A549 cells	Lysosomal dysfunction and cytoskeleton disruption	Apoptosis	99
MWCNT	24-26 nm diameter, 1.7-6.4 μm length (TEM); 8 mV; nanotube	--	10 and 50 μg·mL^-1^	6 h	RAW 264.7 cells	Lysosomal dysfunction	Cell death	100
GO	200 nm diameter, 0.6-1.0 nm thickness (AFM); -30 mV; nanosheet	--	60 μg·mL^-1^	24, 48, and 72 h	PC12 cells	--	Cell survival	102
GO	390 nm or 66 nm diameter, 1 nm thickness (AFM); 30mV; nanosheet	--	25 μg·mL^-1^	24 h	HUVECs	Increasing intracellular calcium ion (Ca^2+^) level	Apoptosis	103
NDs	119 nm (DLS); -25 mV; irregular shape	Ubiquitin K63	50 μg·mL^-1^	12, 24, and 48 h	A549 cells	Ubiquitination	Cell survival	98
NDs	2-10 nm (TEM); aggregates (40-200 nm)	--	50 μg·mL^-1^	48 h	HepG2 cells	--	Cell death	110
SiNPs	62 nm (TEM); -44 mV; near spherical	--	25, 50, 75, and 100 μg·mL^-1^	24 h	HUVECs	upregulation of MAPK/Erk1/2/mTOR signaling and PI3K/Akt/mTOR signaling pathways	Disturb the cell homeostasis and impair angiogenesis	118
SiNPs	58 nm (TEM); -39 mV; near spherical	--	50 and 100 μg·mL^-1^	3, 6, 12, and 24 h	L-02 and HepG2 cells	Lysosome impairment	Cell death	122
CoFe_2_O_4_ @silica	50 nm; -28 mV; near spherical	Silica caped and PEGylated	60 and 100 μg·mL^-1^	15, 30, 45, and 60 mins, 72 h	MC3T3-E1 cells	ERK1/2 signaling activation	Stimulated *in vitro* differentiation and mineralization of osteoblasts	124
Cd-based QDs	10 nm (TEM); --; --	ZnS caped and carboxyl	10 and 20 nM	6h, 24 h	Mouse renal adenocarcinoma cells,	Oxidative stress	Promoted cell survival	130
CdSe QDs	5 nm (TEM); --; --	ZnS caped and streptavidin	10 nM (*in vitro*); 20 nM (*in vivo*)	24 h (*in vitro*); 2 h (*in vivo*)	Primary hippocampal neurons and Wistar rats	--	Synaptic dysfunction *in vivo*	131
Nd_2_O_3_ NPs	80 nm; --; --	--	45 μg·mL^-1^	2 d	NCI-H460 cells	--	S-phase cell cycle arrest, cell death	137
CeO_2_ NPs	4.3 nm (TEM); -2 to -14 mV; near spherical	GlcNAc, PEG, and PVP	100 ppm	24 h	Late infantile neuronal ceroid lipofuscinosis fibroblasts	Activation of TFEB	Cell survival	138
La_2_O_3_	26 nm; 28 mV; sub-micro aggregates	--	50 μg·mL^-1^	24 h	THP-1 cells	Lysosomal dysfunction	Disrupted homeostatic regulation of activated NLRP3 complexes	142
Eu^III^(OH)_3_ nanorods	80-160 nm length, 25-40 nm diameter (TEM); nanorod	--	50 μg·mL^-1^	24 h	GFP-Htt(Q74) Neuro 2a and Htt(Q74) PC12 cells	--	Cell survival	143
ZnO NPs	<50 nm; -11.5 mV; sub-micro aggregates	--	30 μg·mL^-1^	24 h	A549 cells	Mitochondria damage, lysosome dysfunction and excessive ROS generation	Cell death	151
TiO_2_ NPs	15 nm, 50 nm, and 100nm; < -15 mV; sub-micro aggregates	--	100 μg·mL^-1^	72 h	H4/a-syn-GFP	Activation of TFEB	Reduced clearance of autophagic cargo (α-synuclein)	154
Al_2_O_3_ NPs	8-12 nm; sub-micro aggregates	--	0.01, 0.1, 1, and 10 μg·mL^-1^ (*in vitro*); 1.25 mg·kg^-1^ (*in vivo*)	24 h* in vitro*, 1, 3, 5, and 30 d *in vivo*	HCMECs/D3 cell and C57BL/6 mice	--	Neurovascular toxicity	155

**Notes:** DLS: dynamic light scattering; TEM: transmission electron microscope; AFM: atomic force microscopy; SEM: scanning electron microscopy; TFEB: transcription EB; TLR: toll-like receptor; Zeta Pot.: Zeta Potential; IONPs: iron oxide nanoparticles; LC3-Ⅰ/Ⅱ: LC3-Ⅰ to LC3-Ⅱ conversion; MWCNT: multi-walled carbon nanotube; GO: graphene oxide; NDs: nanodiamonds

## Abbreviations

AKT: protein kinase B
AMPK: AMP-activated protein kinase
ATF6: transcription factor 6
Atg14: autophagy-related protein14
Bcl-2: B-cell lymphoma 2
CTAB: cetyltrimethylammonium bromide
DA: dopamine
DA-PAA-PEG: dopamine-polyacrylic acid- polyethylene glycol
DMSA: dimercaptosuccinic acid
EIF2AK3: eukaryotic translation initiation factor 2 alpha kinase 3
ERK: extracellular signal-regulated kinase
GlcNAc: N-acetyl-glucosamine
JNK: c-jun N-terminal kinase
LC3: microtubule-associated protein 1 light chain3
MEK: mitogen-activated protein kinase
mTOR: mammalian target of rapamycin
MWCNT: multi-walled carbon nanotube
NO: nitric oxide
NOS: nitric oxide synthase
PEG: polyethylene glycol
PI3K: phosphoinositide 3-kinase
PtdIns3K: phosphatidylinositol 3-kinase
PVP: polyvinylpyrrolidone
RANKL: receptor activation of nuclear factor (NF)-κB
RPS6KB: ribosomal protein S6 kinase
TRAIL: tumor necrosis factor-related apoptosis-inducing ligand
TSC2: tuberous Sclerosis Complex 2
UPR: unfolded protein response
VPS34: phosphatidylinositol 3-kinase

## References

[1] Rauscher H, Sokull-Klüttgen B, Stamm H (2013). The European Commission's recommendation on the definition of nanomaterial makes an impact. Nanotoxicology.

[2] Akbarzadeh A, Samiei M, Davaran S (2012). Magnetic nanoparticles: preparation, physical properties, and applications in biomedicine. Nanoscale Res Lett.

[3] Murphy CJ, Gole AM, Hunyadi SE, Stone JW, Sisco PN, Alkilany A (2008). Chemical sensing and imaging with metallic nanorods. Chem Commun.

[4] Chen HHC, Yu C, Ueng TH, Chen S, Chen BJ, Huang KJ (1998). Acute and subacute toxicity study of water-soluble Polyalkylsulfonated C60 in rats. Toxicol Pathol.

[5] Stern ST, Zolnik BS, McLeland CB, Clogston J, Zheng J, McNeil SE (2008). Induction of autophagy in porcine kidney cells by quantum dots: A common cellular response to nanomaterials. Toxicol Sci.

[6] Muthu MS, Leong DT, Mei L, Feng S (2014). Nanotheranostics ˗ Application and further development of nanomedicine strategies for advanced theranostics. Theranostics.

[7] Fan W, Yung B, Huang P, Chen X (2017). Nanotechnology for multimodal synergistic cancer therapy. Chem Rev.

[8] Lu J, Chen Z, Pan F, Curtiss LA, Amine K (2016). The role of nanotechnology in the development of battery materials for electric vehicles. Nat Nanotechnol.

[9] Kang MH, Das J, Gurunathan S, Park HW, Song H, Park C (2017). The cytotoxic effects of dimethyl sulfoxide in mouse preimplantation embryos: A mechanistic study. Theranostics.

[10] Li P, Ma R, Dong L, Liu L, Zhou G, Tian Z (2019). Autophagy impairment contributes to PBDE-47-induced developmental neurotoxicity and its relationship with apoptosis. Theranostics.

[11] Li C, Liu H, Sun Y, Wang H, Guo F, Rao S (2009). PAMAM nanoparticles promote acute lung injury by inducing autophagic cell death through the Akt-TSC2-mTOR signaling pathway. J Mol Cell Biol.

[12] Wang F, Bexiga MG, Anguissola S, Boya P, Simpson JC, Salvati A (2013). Time resolved study of cell death mechanisms induced by amine-modified polystyrene nanoparticles. Nanoscale.

[13] Chiu HW, Xia T, Lee YH, Chen CW, Tsai JC, Wang YJ (2015). Cationic polystyrene nanospheres induce autophagic cell death through the induction of endoplasmic reticulum stress. Nanoscale.

[14] Li Y, Zhu H, Wang S, Qian X, Fan J, Wang Z (2015). Interplay of oxidative stress and autophagy in PAMAM dendrimers-induced neuronal cell death. Theranostics.

[15] Zhang X, Yang Y, Liang X, Zeng X, Liu Z, Tao W (2014). Enhancing therapeutic effects of docetaxel-loaded dendritic copolymer nanoparticles by co-treatment with autophagy inhibitor on breast cancer. Theranostics.

[16] Zhang J, Zhang X, Liu G, Chang D, Liang X, Zhu X (2016). Intracellular trafficking network of protein nanocapsules: endocytosis, exocytosis and autophagy. Theranostics.

[17] Mao BH, Tsai JC, Chen CW, Yan SJ, Wang YJ (2016). Mechanisms of silver nanoparticle-induced toxicity and important role of autophagy. Nanotoxicology.

[18] Stern ST, Adiseshaiah PP, Crist RM (2012). Autophagy and lysosomal dysfunction as emerging mechanisms of nanomaterial toxicity. Part Fibre Toxicol.

[19] Qi M, Zhang L, Ma Y, Shuai Y, Li L, Luo K (2017). Autophagy maintains the function of bone marrow mesenchymal stem cells to prevent estrogen deficiency-induced osteoporosis. Theranostics.

[20] Jiang S, Li T, Ji T, Yi W, Yang Z, Wang S (2018). AMPK: Potential therapeutic target for ischemic stroke. Theranostics.

[21] Pandit R, Leinenga G, Götz J (2019). Repeated ultrasound treatment of tau transgenic mice clears neuronal tau by autophagy and improves behavioral functions. Theranostics.

[22] Mo R, Lai R, Lu J, Zhuang Y, Zhou T, Jiang S (2018). Enhanced autophagy contributes to protective effects of IL-22 against acetaminophen-induced liver injury. Theranostics.

[23] Zheng K, He Z, Kitazato K, Wang Y (2019). Selective autophagy regulates cell cycle in cancer therapy. Theranostics.

[24] Roy M, Liang L, Xiao X, Peng Y, Luo Y, Zhou W (2016). Lycorine downregulates HMGB1 to inhibit autophagy and enhances bortezomib activity in multiple myeloma. Theranostics.

[25] Zhou F, Yang X, Zhao H, Liu Y, Feng Y, An R (2018). Down-regulation of OGT promotes cisplatin resistance by inducing autophagy in ovarian cancer. Theranostics.

[26] Sun Y, Huang YH, Huang FY, Mei WL, Liu Q, Wang CC (2018). et al. 3'-epi-12β-hydroxyfroside, a new cardenolide, induces cytoprotective autophagy via blocking the Hsp90/Akt/mTOR axis in lung cancer cells. Theranostics.

[27] Zhang Z, Gao W, Zhou L, Chen Y, Qin S, Zhang L (2019). Repurposing brigatinib for the treatment of colorectal cancer based on inhibition of ER-phagy. Theranostics.

[28] Laurent S, Forge D, Port M, Roch A, Robic C, Elst LV, Muller RN (2008). Magnetic iron oxide nanoparticles: Synthesis, stabilization, vectorization, physicochemical characterizations, and biological applications. Chem Rev.

[29] Ulbrich K, Holá K, Šubr V, Bakandritsos A, Tuček J, Zbořil R (2016). Targeted drug delivery with polymers and magnetic nanoparticles: Covalent and noncovalent approaches, release control, and clinical studies. Chem Rev.

[30] Daldrup-Link HE (2017). Ten things you might not know about iron oxide nanoparticles. Radiology.

[31] Barrow M, Taylor A, Murray P, Rosseinsky MJ, Adams DJ (2015). Design considerations for the synthesis of polymer coated iron oxide nanoparticles for stem cell labelling and tracking using MRI. Chem Soc Rev.

[32] Khan MI, Mohammad A, Patil G, Naqvi SAH, Chauhan LKS, Ahmad I (2012). Induction of ROS, mitochondrial damage and autophagy in lung epithelial cancer cells by iron oxide nanoparticles. Biomaterials.

[33] Park EJ, Choi DH, Kim Y, Lee EW, Song J, Cho MH, Kim JH, Kim SW (2014). Magnetic iron oxide nanoparticles induce autophagy preceding apoptosis through mitochondrial damage and ER stress in RAW264.7 cells. Toxicol In Vitro.

[34] Wang L, Wang Z, Li X, Zhang Y, Yin M, Li J (2018). Deciphering active biocompatibility of iron oxide nanoparticles from their intrinsic antagonism. Nano Res.

[35] Lee J, Giordano S, Zhang J (2012). Autophagy, mitochondria and oxidative stress: Cross-talk and redox signalling. Biochem J.

[36] Huang D, Zhou H, Gao J (2015). Nanoparticles modulate autophagic effect in a dispersity-dependent manner. Sci Rep.

[37] Xu Y, Li Y, Liu X, Pan Y, Sun Z, Xue Y (2019). SPIONs enhances IL-10-producing macrophages to relieve sepsis via Cav1-Notch1/HES1-mediated autophagy.

[38] Shi M, Cheng L, Zhang Z, Liu Z, Mao X (2015). Ferroferric oxide nanoparticles induce prosurvival autophagy in human blood cells by modulating the Beclin 1/Bcl-2/VPs34 complex. Int J Nanomedicine.

[39] Jin R, Liu L, Zhu W, Li D, Yang L, Duan J (2019). Iron oxide nanoparticles promote macrophage autophagy and inflammatory response through activation of toll-like Receptor-4 signaling. Biomaterials.

[40] Zhang X, Zhang H, Liang X, Zhang J, Tao W, Zhu X (2016). Iron oxide nanoparticles induce autophagosome accumulation through multiple mechanisms: Lysosome impairment, mitochondrial damage, and ER stress. Mol Pharm.

[41] Wu QH, Jin RR, Feng T, Liu L, Yang L, Tao Y.H (2017). Iron oxide nanoparticles and induced autophagy in human monocytes. Int J Nanomedicine.

[42] Feng Q, Liu Y, Huang J, Chen K, Huang J, Xiao K (2018). Uptake, distribution, clearance, and toxicity of iron oxide nanoparticles with different sizes and coatings. Sci Rep.

[43] Sadhukha T, Wiedmann TS, Panyam J (2014). Enhancing therapeutic efficacy through designed aggregation of nanoparticles. Biomaterials.

[44] Park EJ, Umh HN, Choi DH, Cho MH, Choi W, Kim SW (2014). Magnetite- and maghemite-induced different toxicity in murine alveolar macrophage cells. Arch Toxicol.

[45] Wu YN, Yang LX, Shi XY, Li IC, Biazik JM, Ratinac KR (2011). The selective growth inhibition of oral cancer by iron core-gold shell nanoparticles through mitochondria-mediated autophagy. Biomaterials.

[46] Wang G, Qian K, Mei X (2018). A theranostic nanoplatform: magneto-gold@fluorescence polymer nanoparticles for tumor targeting T-1&T-2-MRI/CT/NIR fluorescence imaging and induction of genuine autophagy mediated chemotherapy. Nanoscale.

[47] Halamoda Kenzaoui B, Chapuis Bernasconi C, Guney-Ayra S, Juillerat-Jeanneret L (2012). Induction of oxidative stress, lysosome activation and autophagy by nanoparticles in human brain-derived endothelial cells. Biochem J.

[48] Du S, Li J, Du C, Huang Z, Chen G, Yan W (2017). Overendocytosis of superparamagnetic iron oxide particles increases apoptosis and triggers autophagic cell death in human osteosarcoma cell under a spinning magnetic field. Oncotarget.

[49] Shen T, Zhu W, Yang L, Liu L, Jin R, Duan J (2018). Lactosylated N-Alkyl polyethylenimine coated iron oxide nanoparticles induced autophagy in mouse dendritic cells. Regen Biomater.

[50] Ahamed M, AlSalhi MS, Siddiqui MKJ (2010). Silver nanoparticle applications and human health. Clin Chim Acta.

[51] Sun Y (2010). Silver nanowires - Unique templates for functional nanostructures. Nanoscale.

[52] Van De Groep J, Spinelli P, Polman A (2012). Transparent conducting silver nanowire networks. Nano Lett.

[53] Xu R, Ma J, Sun X, Chen Z, Jiang X, Guo Z (2009). Ag nanoparticles sensitize IR-induced killing of cancer cells. Cell Res.

[54] Liu P, Huang Z, Chen Z, Xu R, Wu H, Zang F (2013). Silver nanoparticles: A novel radiation sensitizer for glioma. Nanoscale.

[55] Choi O, Hu Z (2008). Size dependent and reactive oxygen species related nanosilver toxicity to nitrifying bacteria. Environ Sci Technol.

[56] Wu H, Lin J, Liu P, Huang Z, Zhao P, Jin H (2016). Reactive oxygen species acts as executor in radiation enhancement and autophagy inducing by AgNPs. Biomaterials.

[57] Zhu L, Guo D, Sun L, Huang Z, Zhang X, Ma W (2017). Activation of autophagy by elevated reactive oxygen species rather than released silver ions promotes cytotoxicity of polyvinylpyrrolidone-coated silver nanoparticles in hematopoietic cells. Nanoscale.

[58] Xu Y, Wang L, Bai R, Zhang T, Chen C (2015). Silver nanoparticles impede phorbol myristate acetate-induced monocyte-macrophage differentiation and autophagy. Nanoscale.

[59] Miyayama T, Fujiki K, Matsuoka M (2018). Silver nanoparticles induce lysosomal-autophagic defects and decreased expression of transcription factor EB in A549 human lung adenocarcinoma cells. Toxicol In Vitro.

[60] Villeret B, Dieu A, Straube M, Solhonne B, Miklavc P, Hamadi S (2018). Silver nanoparticles impair retinoic acid-inducible gene I-mediated mitochondrial antiviral immunity by blocking the autophagic flux in lung epithelial cells. ACS Nano.

[61] Wu H, Lin J, Liu P, Huang Z, Zhao P, Jin H (2015). Is the autophagy a friend or foe in the silver nanoparticles associated radiotherapy for glioma?. Biomaterials.

[62] Lin J, Huang Z, Wu H, Zhou W, Jin P, Wei P (2014). Inhibition of autophagy enhances the anticancer activity of silver nanoparticles. Autophagy.

[63] Lee TY, Liu MS, Huang LJ, Lue SI, Lin LC, Kwan AL (2013). Bioenergetic failure correlates with autophagy and apoptosis in rat liver following silver nanoparticle intraperitoneal administration. Part Fibre Toxicol.

[64] Lin J, Liu Y, Wu H, Huang Z, Ma J, Guo C (2018). Key role of TFEB nucleus translocation for silver nanoparticle-induced cytoprotective autophagy. Small.

[65] Mishra AR, Zheng J, Tang X, Goering PL (2016). Silver nanoparticle-induced autophagic-Lysosomal disruption and NLRP3-inflammasome activation in HepG2 cells is size-dependent. Toxicol Sci.

[66] Maiuri MC, Zalckvar E, Kimchi A, Kroemer G (2007). Self-eating and self-killing: Crosstalk between autophagy and apoptosis. Nat Rev Mol Cell Biol.

[67] Verma NK, Conroy J, Lyons PE, Coleman J, O'Sullivan MP, Kornfeld H (2012). Autophagy induction by silver nanowires: A new aspect in the biocompatibility assessment of nanocomposite thin films. Toxicol Appl Pharmacol.

[68] Maynard AD, Aitken RJ, Butz T, Colvin V, Donaldson K, Oberdörster G (2006). Safe handling of nanotechnology. Nature.

[69] Lowry GV, Gregory KB, Apte SC, Lead JR (2012). Guest comment: Transformations of nanomaterials in the environment focus issue. Environ Sci Technol.

[70] Lee YH, Cheng FY, Chiu HW, Tsai JC, Fang CY, Chen CW (2014). Cytotoxicity, oxidative stress, apoptosis and the autophagic effects of silver nanoparticles in mouse embryonic fibroblasts. Biomaterials.

[71] Zhao X, Qi T (2018). Kong C, Hao M, Wang Y, Li J, et al. Photothermal exposure of polydopamine-coated branched Au - Ag nanoparticles induces cell cycle arrest, apoptosis, and autophagy in human bladder cancer cells. Int J Nanomedicine.

[72] Zarska M, Sramek M, Novotny F, Havel F, Babelova A, Mrazkova B (2018). Biological safety and tissue distribution of (16-mercaptohexadecyl)trimethylammonium bromide-modified cationic gold nanorods. Biomaterials.

[73] Huang X, El-Sayed IH, Qian W, El-Sayed MA (2006). Cancer cell imaging and photothermal therapy in the near-infrared region by using gold nanorods. J Am Chem Soc.

[74] Qian J, Jiang L, Cai F, Wang D, He S (2011). Fluorescence-surface enhanced Raman scattering co-functionalized gold nanorods as near-infrared probes for purely optical in vivo imaging. Biomaterials.

[75] Wijaya A, Schaffer SB, Pallares IG, Hamad-Schifferli K (2009). Selective release of multiple DNA oligonucleotides from gold nanorods. ACS Nano.

[76] Manshian BB, Moyano DF, Corthout N, Munck S, Himmelreich U, Rotello VM (2014). High-content imaging and gene expression analysis to study cell-nanomaterial interactions: The effect of surface hydrophobicity. Biomaterials.

[77] Wan J, Wang JH, Liu T, Xie Z, Yu XF, Li W (2015). Surface chemistry but not aspect ratio mediates the biological toxicity of gold nanorods in vitro and in vivo. Sci Rep.

[78] Lu HY, Chang YJ, Fan NC, Wang LS, Lai NC, Yang CM (2015). Synergism through combination of chemotherapy and oxidative stress-induced autophagy in A549 lung cancer cells using redox-responsive nanohybrids: A new strategy for cancer therapy. Biomaterials.

[79] Zhang Y, Kong N, Zhang Y, Yang W, Yan F (2017). Size-dependent effects of gold nanoparticles on osteogenic differentiation of human periodontal ligament progenitor cells. Theranostics.

[80] Ke S, Zhou T, Yang P, Wang Y, Zhang P, Chen K (2017). Gold nanoparticles enhance TRAIL sensitivity through Drpl-mediated apoptotic and autophagic mitochondrial fission in NSCLC cells. Int J Nanomedicine.

[81] Ma X, Wu Y, Jin S, Tian Y, Zhang X, Zhao Y (2011). Gold nanoparticles induce autophagosome accumulation through size-dependent nanoparticle uptake and lysosome impairment. ACS Nano.

[82] Li JJ, Hartono D, Ong CN, Bay BH, Yung LYL (2010). Autophagy and oxidative stress associated with gold nanoparticles. Biomaterials.

[83] Bosi S, Da Ros T, Spalluto G, Prato M (2003). Fullerene derivatives: An attractive tool for biological applications. Eur J Med Chem.

[84] Johnson-Lyles DN, Peifley K, Lockett S, Neun BW, Hansen M, Clogston J (2010). Fullerenol cytotoxicity in kidney cells is associated with cytoskeleton disruption, autophagic vacuole accumulation, and mitochondrial dysfunction. Toxicol Appl Pharmacol.

[85] Orecna M, De Paoli SH, Janouskova O, Tegegn TZ, Filipova M, Bonevich JE (2014). Toxicity of carboxylated carbon nanotubes in endothelial cells is attenuated by stimulation of the autophagic flux with the release of nanomaterial in autophagic vesicles. Nanomedicine.

[86] Choi KY, Liu G, Lee S, Chen X (2012). Theranostic nanoplatforms for simultaneous cancer imaging and therapy: Current approaches and future perspectives. Nanoscale.

[87] Wang Y, Li Z, Hu D, Lin CT, Li J, Lin Y (2010). Aptamer/graphene oxide nanocomplex for in situ molecular probing in living cells. J Am Chem Soc.

[88] Kim H, Namgung R, Singha K, Oh IK, Kim WJ (2011). Graphene oxide-polyethylenimine nanoconstruct as a gene delivery vector and bioimaging tool. Bioconjug Chem.

[89] Zhang L, Lu Z, Zhao Q, Huang J, Shen H, Zhang Z (2011). Enhanced chemotherapy efficacy by sequential delivery of siRNA and anticancer drugs using PEI-grafted graphene oxide. Small.

[90] Chen GY, Yang HJ, Lu CH, Chao YC, Hwang SM, Chen CL (2012). Simultaneous induction of autophagy and toll-like receptor signaling pathways by graphene oxide. Biomaterials.

[91] Chen GY, Pang DWP, Hwang SM, Tuan HY, Hu YC (2012). A graphene-based platform for induced pluripotent stem cells culture and differentiation. Biomaterials.

[92] Yang K, Wan J, Zhang S, Tian B, Zhang Y, Liu Z (2012). The influence of surface chemistry and size of nanoscale graphene oxide on photothermal therapy of cancer using ultra-low laser power. Biomaterials.

[93] Mochalin VN, Shenderova O, Ho D, Gogotsi Y (2012). The properties and applications of nanodiamonds. Nat Nanotechnol.

[94] Jeong JK, Lee YJ, Jeong SY, Jeong S, Lee GW, Park SY (2017). Autophagic flux induced by graphene oxide has a neuroprotective effect against human prion protein fragments. Int J Nanomedicine.

[95] Park EJ, Zahari NEM, Kang MS, Lee SJ, Lee K, Lee BS (2014). Toxic response of HIPCO single-walled carbon nanotubes in mice and RAW264.7 macrophage cells. Toxicol Lett.

[96] Yamawaki H, Iwai N (2006). Cytotoxicity of water-soluble fullerene in vascular endothelial cells. Am J Physiol Cell Physiol.

[97] Zhang Q, Yang W, Man N, Zheng F, Shen Y, Sun K (2009). Autophagy-mediated chemosensitization in cancer cells by fullerene C60 nanocrystal. Autophagy.

[98] Liu KK, Qiu WR, Naveen Raj E, Liu HF, Huang HS, Lin YW (2017). Ubiquitin-coated nanodiamonds bind to autophagy receptors for entry into the selective autophagy pathway. Autophagy.

[99] Mittal S, Sharma PK, Tiwari R, Rayavarapu RG, Shankar J, Chauhan LKS (2017). Impaired lysosomal activity mediated autophagic flux disruption by graphite carbon nanofibers induce apoptosis in human lung epithelial cells through oxidative stress and energetic impairment. Part Fibre Toxicol.

[100] Cohignac V, Landry MJ, Ridoux A, Pinault M, Annangi B, Gerdil A (2018). Carbon nanotubes, but not spherical nanoparticles, block autophagy by a shape-related targeting of lysosomes in murine macrophages. Autophagy.

[101] Liu HL, Zhang YL, Yang N, Zhang YX, Liu XQ, Li CG (2011). A functionalized single-walled carbon nanotube-induced autophagic cell death in human lung cells through Akt-TSC2-mTOR signaling. Cell Death Dis.

[102] Jin P, Wei P, Zhang Y, Lin J, Sha R, Hu Y (2016). Autophagy-mediated clearance of ubiquitinated mutant huntingtin by graphene oxide. Nanoscale.

[103] Lim MH, Jeung IC, Jeong J, Yoon SJ, Lee SH, Park J (2016). Graphene oxide induces apoptotic cell death in endothelial cells by activating autophagy via calcium-dependent phosphorylation of c-Jun N-terminal kinases. Acta Biomater.

[104] Qin C, Liu Q, Hu ZW, Zhou LQ, Shang K, Bosco DB (2018). Microglial TLR4-dependent autophagy induces ischemic white matter damage via STAT1/6 pathway. Theranostics.

[105] Binotti B, Pavlos NJ, Riedel D, Wenzel D, Schalk AM, Karin K (2015). The GTPase Rab26 links synaptic vesicles to the autophagy pathway. Elife.

[106] Li H, He B, Liu X, Li J, Liu Q, Dong W (2017). Regulation on toll-like receptor 4 and cell barrier function by Rab26 siRNA-loaded DNA nanovector in pulmonary microvascular endothelial cells. Theranostics.

[107] Park EJ, Zahari NEM, Lee EW, Song J, Lee JH, Cho MH (2014). SWCNTs induced autophagic cell death in human bronchial epithelial cells. Toxicol In Vitro.

[108] Wu L, Zhang Y, Zhang C, Cui X, Zhai S, Liu Y (2014). Tuning cell autophagy by diversifying carbon nanotube surface chemistry. ACS Nano.

[109] Xu J, Wang H, Hu Y, Zhang YS, Wen L, Yin F (2019). Inhibition of CaMKII alpha activity enhances antitumor effect of fullerene C60 nanocrystals by suppression of autophagic degradation. Adv Sci.

[110] Cui Z, Zhang Y, Xia K, Yan Q, Kong H, Zhang J (2018). Nanodiamond autophagy inhibitor allosterically improves the arsenical-based therapy of solid tumors. Nat Commun.

[111] Chen M, Von Mikecz A (2005). Formation of nucleoplasmic protein aggregates impairs nuclear function in response to SiO2 nanoparticles. Exp Cell Res.

[112] He Q, Shi J, Chen F, Zhu M, Zhang L (2010). An anticancer drug delivery system based on surfactant-templated mesoporous silica nanoparticles. Biomaterials.

[113] He Q, Shi J (2011). Mesoporous silica nanoparticle based nano drug delivery systems : synthesis, controlled drug release and delivery, pharmacokinetics and biocompatibility. J Mater Chem.

[114] Zhang Z, Wang L, Wang J, Jiang X, Li X, Hu Z (2012). Mesoporous silica-coated gold nanorods as a light-mediated multifunctional theranostic platform for cancer treatment. Adv Mater.

[115] Xie M, Shi H, Ma K, Shen H, Li B, Shen S (2013). Hybrid nanoparticles for drug delivery and bioimaging: Mesoporous silica nanoparticles functionalized with carboxyl groups and a near-infrared fluorescent dye. J Colloid Interface Sci.

[116] Wang Y, Zhao Q, Han N, Bai L, Li J, Liu J (2015). Mesoporous silica nanoparticles in drug delivery and biomedical applications. Nanomedicine.

[117] Du S, Li C, Lu Y, Lei X, Zhang Y, Li S (2019). Dioscin alleviates crystalline silica-induced pulmonary inflammation and fibrosis through promoting alveolar macrophage autophagy. Theranostics.

[118] Duan J, Yu Y, Yu Y, Li Y, Huang P, Zhou X (2014). Silica nanoparticles enhance autophagic activity, disturb endothelial cell homeostasis and impair angiogenesis. Part Fibre Toxicol.

[119] Guo C, Yang M, Jing L, Wang J, Yu Y, Li Y (2016). Amorphous silica nanoparticles trigger vascular endothelial cell injury through apoptosis and autophagy via reactive oxygen species-mediated MAPK/Bcl-2 and PI3K/Akt/mTORsignaling. Int J Nanomedicine.

[120] Wang J, Li Y, Duan J, Yang M, Yu Y, Feng L (2018). Silica nanoparticles induce autophagosome accumulation via activation of the EIF2AK3 and ATF6 UPR pathways in hepatocytes. Autophagy.

[121] Manshian BB, Himmelreich U, Soenen SJ (2017). Standard cellular testing conditions generate an exaggerated nanoparticle cytotoxicity profile. Chem Res Toxicol.

[122] Wang J, Yu Y, Lu K, Yang M, Li Y, Zhou X (2017). Silica nanoparticles induce autophagy dysfunction via lysosomal impairment and inhibition of autophagosome degradation in hepatocytes. Int J Nanomedicine.

[123] Duan J, Yu Y, Yu Y, Li Y, Wang J, Geng W (2014). Silica nanoparticles induce autophagy and endothelial dysfunction via the PI3K/Akt/mTOR signaling pathway. Int J Nanomedicine.

[124] Ha SW, Neale Weitzmann M, Beck GR (2014). Bioactive silica nanoparticles promote osteoblast differentiation through stimulation of autophagy and direct association with LC3 and p62. ACS Nano.

[125] Li X, He Q, Shi J (2014). Global gene expression analysis of cellular death mechanisms induced by mesoporous silica nanoparticle-based drug delivery system. ACS Nano.

[126] De La Zerda A, Gambhir SS (2007). Drug delivery: Keeping tabs on nanocarriers. Nat Nanotechnol.

[127] Michalet X, Pinaud FF, Bentolila LA, Tsay JM, Doose S, Li JJ (2005). Quantum dots for live cells, in vivo imaging, and diagnostics. Science.

[128] Lovrić J, Cho SJ, Winnik FM, Maysinger D (2005). Unmodified cadmium telluride quantum dots induce reactive oxygen species formation leading to multiple organelle damage and cell death. Chem Biol.

[129] Ipe BI, Lehnig M, Niemeyer CM (2005). On the generation of free radical species from quantum dots. Small.

[130] Luo YH, Wu SB, Wei YH, Chen YC, Tsai MH, Ho CC (2013). Cadmium-based quantum dot induced autophagy formation for cell survival via oxidative stress. Chem Res Toxicol.

[131] Chen L, Miao Y, Chen L, Jin P, Zha Y, Chai Y (2013). The role of elevated autophagy on the synaptic plasticity impairment caused by CdSe/ZnS quantum dots. Biomaterials.

[132] Chen J, Patil S, Seal S, McGinnis JF (2006). Rare earth nanoparticles prevent retinal degeneration induced by intracellular peroxides. Nat Nanotechnol.

[133] Tarnuzzer RW, Colon J, Patil S, Seal S (2005). Vacancy engineered ceria nanostructures for protection from radiation-induced cellular damage. Nano Lett.

[134] Das M, Patil S, Bhargava N, Kang JF, Riedel LM, Seal S (2007). Auto-catalytic ceria nanoparticles offer neuroprotection to adult rat spinal cord neurons. Biomaterials.

[135] Korsvik C, Patil S, Seal S, Self WT (2007). Superoxide dismutase mimetic properties exhibited by vacancy engineered ceria nanoparticles.

[136] Hussain S, Al-Nsour F, Rice AB, Marshburn J, Yingling B, Ji Z (2012). Cerium dioxide nanoparticles induce apoptosis and autophagy in human peripheral blood monocytes. ACS Nano.

[137] Chen Y, Yang L, Feng C, Wen LP (2005). Nano neodymium oxide induces massive vacuolization and autophagic cell death in non-small cell lung cancer NCI-H460 cells. Biochem Biophys Res Commun.

[138] Song W, Soo Lee S, Savini M, Popp L, Colvin VL, Segatori L (2014). Ceria nanoparticles stabilized by organic surface coatings activate the lysosome-autophagy system and enhance autophagic clearance. ACS Nano.

[139] Zhang Y, Zheng F, Yang T, Zhou W, Liu Y, Man N (2012). Tuning the autophagy-inducing activity of lanthanide-based nanocrystals through specific surface-coating peptides. Nat Mater.

[140] Zhu S, Zhang J, Zhang L, Ma W, Man N, Liu Y (2017). Inhibition of Kupffer cell autophagy abrogates nanoparticle-induced liver injury. Adv Healthc Mater.

[141] Zhang JQ, Zhou W, Zhu SS, Lin J, Wei PF, Li FF (2017). Persistency of enlarged autolysosomes underscores nanoparticle-induced autophagy in hepatocytes. Small.

[142] Li R, Ji Z, Qin H, Kang X, Sun B, Wang M (2014). Interference in autophagosome fusion by rare earth nanoparticles disrupts autophagic flux and regulation of an interleukin-1*β* producing inflammasome. ACS Nano.

[143] Wei PF, Zhang L, Nethi SK, Barui AK, Lin J, Zhou W (2014). Accelerating the clearance of mutant huntingtin protein aggregates through autophagy induction by europium hydroxide nanorods. Biomaterials.

[144] Wei PF, Jin PP, Barui AK, Hu Y, Zhang L, Zhang JQ (2015). Differential ERK activation during autophagy induced by europium hydroxide nanorods and trehalose: Maximum clearance of huntingtin aggregates through combined treatment. Biomaterials.

[145] Rasmussen JW, Martinez E, Louka P, Wingett DG (2010). Zinc oxide nanoparticles for selective destruction of tumor cells and potential for drug delivery applications. Expert Opin Drug Deliv.

[146] Wang J, Lee JS, Kim D, Zhu L (2017). Exploration of zinc oxide nanoparticles as a multitarget and multifunctional anticancer nanomedicine. ACS Appl Mater Interfaces.

[147] Yu KN, Yoon TJ, Minai-Tehrani A, Kim JE, Park SJ, Jeong MS (2013). Zinc oxide nanoparticle induced autophagic cell death and mitochondrial damage via reactive oxygen species generation. Toxicol In Vitro.

[148] Chevallet M, Gallet B, Fuchs A, Jouneau PH, Um K, Mintz E (2016). Metal homeostasis disruption and mitochondrial dysfunction in hepatocytes exposed to sub-toxic doses of zinc oxide nanoparticles. Nanoscale.

[149] Bai DP, Zhang XF, Zhang GL, Huang YF, Gurunathan S (2017). Zinc oxide nanoparticles induce apoptosis and autophagy in human ovarian cancer cells. Int J Nanomedicine.

[150] Hu Y, Zhang HR, Dong L, Xu MR, Zhang L, Ding WP (2019). Enhancing tumor chemotherapy and overcoming drug resistance through autophagy-mediated intracellular dissolution of zinc oxide nanoparticles. Nanoscale.

[151] Zhang J, Qin X, Wang B, Xu G, Qin Z, Wang J (2017). Zinc oxide nanoparticles harness autophagy to induce cell death in lung epithelial cells. Cell Death Dis.

[152] Wang B, Zhang J, Chen C, Xu G, Qin X, Hong Y (2018). The size of zinc oxide nanoparticles controls its toxicity through impairing autophagic flux in A549 lung epithelial cells. Toxicol Lett.

[153] Weir A, Westerhoff P, Fabricius L, Hristovski K, Von Goetz N (2012). Titanium dioxide nanoparticles in food and personal care products. Environ Sci Technol.

[154] Popp L, Tran V, Patel R, Segatori L (2018). Autophagic response to cellular exposure to titanium dioxide nanoparticles. Acta Biomater.

[155] Chen L, Zhang B, Toborek M (2013). Autophagy is involved in nanoalumina-induced cerebrovascular toxicity. Nanomedicine.

[156] Wang Y, Kaur G, Zysk A, Liapis V, Hay S, Santos A (2015). Systematic in vitro nanotoxicity study on anodic alumina nanotubes with engineered aspect ratio: Understanding nanotoxicity by a nanomaterial model. Biomaterials.

[157] Li D, Wang C, Li Z, Wang H, He J, Zhu J (2018). Nano-sized Al2O3 particle-induced autophagy reduces osteolysis in aseptic loosening of total hip arthroplasty by negative feedback regulation of RANKL expression in fibroblasts. Cell Death Dis.

[158] Li H, Li Y, Jiao J, Hu HM (2011). Alpha-alumina nanoparticles induce efficient autophagy-dependent cross-presentation and potent antitumour response. Nat Nanotechnol.

